# Updating the MASH pharmacotherapy landscape: a network meta-analysis incorporating SGLT2 inhibitors and emerging combination therapies

**DOI:** 10.3389/fendo.2026.1829315

**Published:** 2026-06-04

**Authors:** Mengshi Tang, Yingxu Ma, Yating Wang

**Affiliations:** 1 Department of Rheumatology and Immunology, The Second Xiangya Hospital, Central South University, Changsha, Hunan, China; 2 Department of Cardiovascular Medicine, The Second Xiangya Hospital, Central South University, Changsha, Hunan, China

**Keywords:** FGF21 analogs, GLP-1 receptor agonists, liver fibrosis, metabolic dysfunction-associated steatohepatitis (MASH), network meta-analysis, SGLT2 inhibitors

## Abstract

**Background:**

Multiple pharmacological agents targeting distinct pathways (e.g., GLP-1, FGF21, THR-β) show promise for metabolic dysfunction-associated steatohepatitis (MASH). However, while recent meta-analyses have established the efficacy of GLP-1 RAs and FGF21 analogs, they largely predate critical histological trials on SGLT2 inhibitors, leaving the comparative position of this highly accessible oral therapy undefined. We aimed to systematically rank the efficacy and tolerability of distinct pharmacological mechanisms for MASH and characterize their benefit–risk profiles through cluster analysis.

**Methods:**

We searched PubMed, Embase, Web of Science, Scopus, and Cochrane Library for Phase II/III randomized controlled trials (RCTs) involving adults with biopsy-proven MASH. The primary outcome was fibrosis improvement (≥1 stage reduction without MASH worsening). Secondary outcomes included MASH resolution and safety (discontinuation due to adverse events). A frequentist network meta-analysis (NMA) was performed.

**Results:**

We included 34 studies from 33 unique publications. For fibrosis improvement, FGF21 analogs (RR 2.22, 95% CI 1.40-3.54) and SGLT2 inhibitors (RR 2.27, 95% CI 1.22-4.17) ranked highest. Notably, SGLT2 inhibitors numerically outperformed the FDA-approved THR-β agonist (RR 1.61) and GLP-1 RAs (RR 1.51, 95% CI 1.09-2.10). For MASH resolution, GLP-1/GIP dual agonists (RR 5.21) and FGF21 analogs (RR 3.52) showed the highest efficacy, while SGLT2 inhibitors also yielded significant benefits (RR 2.92, 95% CI 1.18-7.26). In the two-dimensional cluster analysis evaluating the balance between fibrosis efficacy and tolerability, SGLT2 inhibitors numerically occupied the “Optimal Zone,” though this finding is based on limited histological data. Sensitivity analyses excluding small-scale studies confirmed the robustness of these rankings.

**Conclusion:**

FGF21 analogs (RR 2.22, 95% CI 1.40-3.54) demonstrated the most robust evidence for fibrosis improvement. Preliminary evidence from a single pivotal trial suggests SGLT2 inhibitors (RR 2.27, 95% CI 1.22-4.17) may represent a promising oral option, though this requires confirmation in adequately powered Phase 3 trials. Further adequately powered Phase 3 trials are warranted to validate the magnitude of these histological benefits.

**Systematic review registration:**

https://www.crd.york.ac.uk/prospero/, identifier CRD420261292638.

## 1 Introduction

Metabolic dysfunction-associated steatohepatitis (MASH) represents the progressive phenotype of metabolic dysfunction-associated steatotic liver disease (MASLD) and has emerged as a critical global public health challenge (1, 2). Liver fibrosis is the strongest predictor of adverse long-term clinical outcomes, including cirrhosis, hepatocellular carcinoma, and liver-related mortality, in patients with MASH (3). Critically, fibrosis regression—even by a single stage—is associated with an over 80% reduction in liver-related adverse events, making the reversal of fibrosis the primary surrogate endpoint for regulatory approval (4).

The pharmacological landscape for MASH has evolved rapidly. A historic milestone was achieved with the recent FDA approval of resmetirom, a thyroid hormone receptor-β (THR-β) agonist, based on robust Phase 3 histological data (5). Concurrently, injectable biologics such as FGF21 analogs (e.g., efruxifermin, pegozafermin) and GLP-1-based polyagonists (e.g., tirzepatide) have demonstrated impressive anti-fibrotic and MASH-resolving efficacy in recent clinical trials (6–8). Furthermore, oral pleiotropic agents, notably SGLT2 inhibitors, are being actively explored for their potential to holistically address the metabolic drivers of liver injury (9).

Despite these rapid advancements, critical gaps in the evidence base remain. While recent high-quality network meta-analyses (NMAs) have successfully ranked established agents like resmetirom and GLP-1 receptor agonists (10, 11), these comprehensive reviews largely predated the availability of critical histological data for SGLT2 inhibitors. Consequently, they excluded emerging evidence, such as the recent multicenter trial by Lin et al. (9), potentially underestimating the role of this highly accessible oral antidiabetic class in fibrosis regression. The relative standing of these generic oral agents against potent novel injectables remains ill-defined.

From a mechanistic perspective, SGLT2 inhibitors are hypothesized to influence fibrosis biology through multiple pathways extending beyond glycemic control: (1) attenuation of hepatic *de novo* lipogenesis via reduced substrate delivery and improved insulin sensitivity; (2) mitigation of oxidative stress through enhanced ketogenesis and fatty acid oxidation; (3) potential modulation of the TGF-β1/Smad signaling pathway, attenuating hepatic stellate cell activation; and (4) reduction of systemic inflammation via weight loss and visceral adiposity reduction. These mechanisms differ from incretin-based therapies, which primarily act through weight-loss-driven substrate reduction, and from hepatocyte-targeted agents like THR-β agonists, which directly modulate hepatic lipid metabolism.

To address these limitations, we conducted a comprehensive NMA to: (1) provide an updated comparative efficacy ranking of pharmacological mechanisms for liver fibrosis improvement and MASH resolution; (2) evaluate tolerability profiles based on treatment discontinuation due to adverse events; and (3) perform a two-dimensional cluster analysis to characterize the benefit–risk balance of each drug class—specifically integrating the latest histological data for SGLT2 inhibitors.

## 2 Methods

### 2.1 Study registration and protocol development

This systematic review and network meta-analysis (NMA) was conducted and reported in strict accordance with the Preferred Reporting Items for Systematic Reviews and Meta-Analyses for Network Meta-Analyses (PRISMA-NMA) statement. The study protocol has been registered in the Prospective Register of Systematic Reviews (PROSPERO) database (CRD420261292638).

#### 2.1.1 Eligibility criteria

The study eligibility criteria were defined based on the PICO(S) framework (Population, Intervention, Comparator, Outcomes, and Study design) as follows:

##### 2.1.1.1 Population

Adult patients (≥18 years) with biopsy-confirmed metabolic dysfunction-associated steatohepatitis (MASH), formerly known as non-alcoholic steatohepatitis (NASH), accompanied by liver fibrosis (stages F0-F4 with predominantly F2-F3).

##### 2.1.1.2 Intervention

Any pharmacological intervention aimed at improving MASH or liver fibrosis. The primary drug classes of interest include but are not limited to: GLP-1 receptor-based agents (mono- or dual-agonists), fibroblast growth factor 21 (FGF21) analogues, thyroid hormone receptor beta (THR-β) agonists, peroxisome proliferator-activated receptor (PPAR) agonists, and galectin-3 inhibitors.

##### 2.1.1.3 Comparator

Placebo. To ensure the connectivity of the evidence network, all included interventions must be connected through this common comparator.

##### 2.1.1.4 Outcomes

###### 2.1.1.4.1 Primary outcome

The proportion of patients achieving an improvement in liver fibrosis, defined as a reduction of at least one stage (≥1) with no worsening of MASH.

###### 2.1.1.4.2 Secondary outcomes

1. The proportion of patients achieving MASH resolution, defined as the absence of steatohepatitis with no worsening of liver fibrosis.
2. The incidence of treatment discontinuation due to adverse events (AEs).

Study Design (S): Phase II or III randomized controlled trials (RCTs).

#### 2.1.2 Exclusion criteria

The following were excluded: (1) non-randomized studies (e.g., observational studies, case reports); (2) reviews, editorials, meta-analyses, and conference abstracts (if sufficient data cannot be obtained); (3) animal or *in vitro* (preclinical) studies; (4) studies without a placebo control group; (5) Phase I clinical trials.

### 2.2 Literature retrieval strategy

Computer searches were conducted in databases including PubMed, Embase, Web of Science, Scopus and Cochrane Library. The search period was from the establishment of the databases to August 2025. The search strategy combined subject terms (such as MeSH, Emtree) and free terms. The search revolved around “disease concepts” (such as “MASH”, “NASH”, “Non-alcoholic Steatohepatitis”), “Liver Fibrosis” and “research design concepts” (such as “Randomized Controlled Trial”, “Placebo”, “Clinical Trial”) were constructed and combined. To ensure the sensitivity of the search, the search terms do not contain specific drug names but are identified during the screening stage. The search strategy was independently developed and implemented by two researchers (Y.M. and M.T.). The detailed search formula is shown in **Table 1**.

**Table 1:** Search strategies for electronic databases.

Database	Search strategy
PubMed	#1 (“Metabolic Dysfunction-Associated Steatohepatitis”[Mesh] OR “Non-alcoholic Steatohepatitis”[Mesh] OR “Liver Fibrosis”[Mesh] OR “Liver Cirrhosis”[Mesh] OR MASH[tiab] OR NASH[tiab] OR “metabolic dysfunction-associated steatohepatitis”[tiab] OR “non-alcoholic steatohepatitis”[tiab] OR “nonalcoholic steatohepatitis”[tiab] OR “fatty liver”[tiab] OR “liver fibrosis”[tiab]) #2 (“Randomized Controlled Trial”[Publication Type] OR “Controlled Clinical Trial”[Publication Type] OR “Randomized Controlled Trial”[Mesh] OR “Controlled Clinical Trial”[Mesh] OR randomi*[tiab] OR placebo*[tiab] OR “clinical trial”[tiab] OR “double-blind”[tiab]) #3 #1 AND #2
Embase	#1 ‘metabolic dysfunction associated steatohepatitis’/exp OR ‘nonalcoholic steatohepatitis’/exp OR ‘liver fibrosis’/exp OR ‘liver cirrhosis’/exp OR MASH:ab,ti OR NASH:ab,ti OR ‘metabolic dysfunction-associated steatohepatitis’:ab,ti OR ‘non-alcoholic steatohepatitis’:ab,ti OR ‘nonalcoholic steatohepatitis’:ab,ti OR ‘fatty liver’:ab,ti OR ‘liver fibrosis’:ab,ti #2 ‘randomized controlled trial’/exp OR ‘controlled clinical trial’/exp OR ‘randomization’/exp OR ‘placebo’/exp OR randomi*:ab,ti OR placebo*:ab,ti OR ‘clinical trial’:ab,ti OR ‘double-blind’:ab,ti OR ‘single-blind’:ab,ti #3 #1 AND #2
Web of Science	#1 TS=(MASH) OR TS=(NASH) OR TS=(“metabolic dysfunction-associated steatohepatitis”) OR TS=(“non-alcoholic steatohepatitis”) OR TS=(“nonalcoholic steatohepatitis”) OR TS=(“fatty liver”) OR TS=(“liver fibrosis”) OR TS=(“liver cirrhosis”) #2 TS=(randomi*) OR TS=(placebo*) OR TS=(“clinical trial”) OR TS=(trial) OR TS=(“controlled trial”) OR TS=(“double blind”) OR TS=(“single blind”) #3 #1 AND #2
Scopus	#1 (TITLE-ABS-KEY(MASH) OR TITLE-ABS-KEY(NASH) OR TITLE-ABS-KEY(“metabolic dysfunction-associated steatohepatitis”) OR TITLE-ABS-KEY(“non-alcoholic steatohepatitis”) OR TITLE-ABS-KEY(“nonalcoholic steatohepatitis”) OR TITLE-ABS-KEY(“fatty liver”) OR TITLE-ABS-KEY(“liver fibrosis”) OR TITLE-ABS-KEY(“liver cirrhosis”)) #2 (TITLE-ABS-KEY(randomi*) OR TITLE-ABS-KEY(placebo*) OR TITLE-ABS-KEY(“clinical trial”) OR TITLE-ABS-KEY(trial) OR TITLE-ABS-KEY(“controlled trial”) OR TITLE-ABS-KEY(“double blind”) OR TITLE-ABS-KEY(“single blind”)) #3 #1 AND #2
Cochrane Library	#1 [mh “Non-alcoholic Fatty Liver Disease”] OR [mh “Liver Cirrhosis”] OR [mh “Fibrosis”] #2 (MASH OR NASH OR “metabolic dysfunction-associated steatohepatitis” OR “non-alcoholic steatohepatitis” OR “nonalcoholic steatohepatitis”):ti,ab,kw #3 (“fatty liver” OR “liver fibrosis” OR “hepatic fibrosis” OR “liver cirrhosis” OR “hepatic cirrhosis” OR steatohepatitis):ti,ab,kw #4 #1 OR #2 OR #3 #5 [mh “Randomized Controlled Trials as Topic”] OR [mh “Randomized Controlled Trial”] OR [mh “Clinical Trials as Topic”] #6 (randomi* OR placebo* OR “clinical trial” OR “controlled trial” OR “double blind” OR “single blind” OR blind* OR RCT):ti,ab,kw #7 #5 OR #6 #8 #4 AND #7

### 2.3 Literature screening and data extraction

All retrieved literature was imported into EndNote, and duplicates were removed. Two independent researchers conducted a two-stage screening process based on titles, abstracts, and full texts. Discrepancies were resolved through consensus or a third-party adjudicator.

To maximize the robustness of the safety and tolerability analysis, trials reporting relevant safety outcomes (i.e., adverse events leading to study discontinuation) but lacking biopsy-proven histological endpoints at the designated follow-up were exclusively incorporated into the safety network. To ensure mechanistic consistency and respond to concerns regarding class purity, we excluded data from dual SGLT1/2 inhibitors (e.g., licogliflozin (12)). The SGLT2 inhibitor node was restricted to selective SGLT2 inhibitors (e.g., dapagliflozin) for both efficacy and tolerability analyses. This methodological approach ensures a broader and more reliable evidence base for the safety profile of the drug class without compromising the strict inclusion criteria for histological efficacy.

### 2.4 Data extraction and quality evaluation

#### 2.4.1 Data extraction

Two researchers (Y.M. and M.T.) independently extracted the information included in the study using a pre-designed standardized Excel data extraction table. The extracted content includes:

#### 2.4.2 Basic research information

First author, publication year, research design (such as Phase II/III, multicenter), sample size, source of research funding.

#### 2.4.3 Baseline characteristics of the patient

Age, gender, BMI, proportion of diabetes mellitus (T2DM), baseline stage of liver fibrosis (proportions of F2, F3, and F4).

#### 2.4.4 Intervention and control information

Drug name, mechanism of action, dosage, treatment duration.

#### 2.4.5 Outcome data

For the above primary and secondary outcomes, the number of respondents (n) and the total number (N) of each group were extracted.

When the data is missing or the report is unclear, we attempted to contact the original author of the study to obtain supplementary information.

#### 2.4.6 Risk of bias assessment

Two researchers independently assessed the Risk of Bias for inclusion in RCTs using the RoB 2 (Risk of Bias 2) tool recommended by Cochrane. The assessment was conducted in five areas: (1) Randomization process; (2) Deviation from the predetermined intervention; (3) Missing outcome data; (4) Outcome measurement; (5) Selectively report results. Each area was rated as “low risk”, “some concern” or “high risk”.

### 2.5 Data analysis

This study conducted a network meta-analysis using R software (mainly netmeta) and Stata software (network command). The primary NMA and ranking (P-scores) were performed using the netmeta package in R; Stata’s network command was used for supplementary consistency checks and funnel plot visualization.

#### 2.5.1 Data merging and modeling

For all binary outcomes (primary and secondary outcomes), the Risk Ratio (RR) and its 95% confidence interval (CI) was used as effect sizes. We adopted a frequentist random-effects model for NMA, which can take into account both intra-study and inter-study heterogeneity simultaneously.

#### 2.5.2 Evidence network diagram

We drew an evidence network diagram to visualize the direct and indirect comparative relationships among all intervention measures. The size of the nodes represents the total sample size, and the thickness of the connection lines represents the number of studies.

#### 2.5.3 Consistency test

To ensure the reliability of NMA results, we assessed the consistency between direct comparison and indirect comparison evidence in the network. The “Node-splitting” model was adopted for local consistency test. If the P value is greater than 0.05, it is considered that the consistency between direct and indirect evidence is good.

#### 2.5.4 NMA results and ranking

Calculate the RR values and 95% CI for all drug pairwise comparisons (including those with placebo). Meanwhile, interventions were ranked using P-scores, which represent the frequentist equivalent to the Surface Under the Cumulative Ranking curve (SUCRA). The P-score ranges from 0 to 1, with values closer to 1 indicating a higher certainty that an intervention is superior to others.

#### 2.5.5 Heterogeneity and publication bias

We assessed the statistical heterogeneity across the entire network. Comparison-adjusted funnel plots were used to evaluate the possible publication bias or small sample effect in the network.

#### 2.5.6 Subgroup analysis

If the data permit, we conducted subgroup analysis based on preset covariates, such as exploring the impact of different baseline liver fibrosis degrees (F2 vs F3) on drug efficacy.

## 3 Results

### 3.1 Literature screening results and evidence network

**Figure 1:**
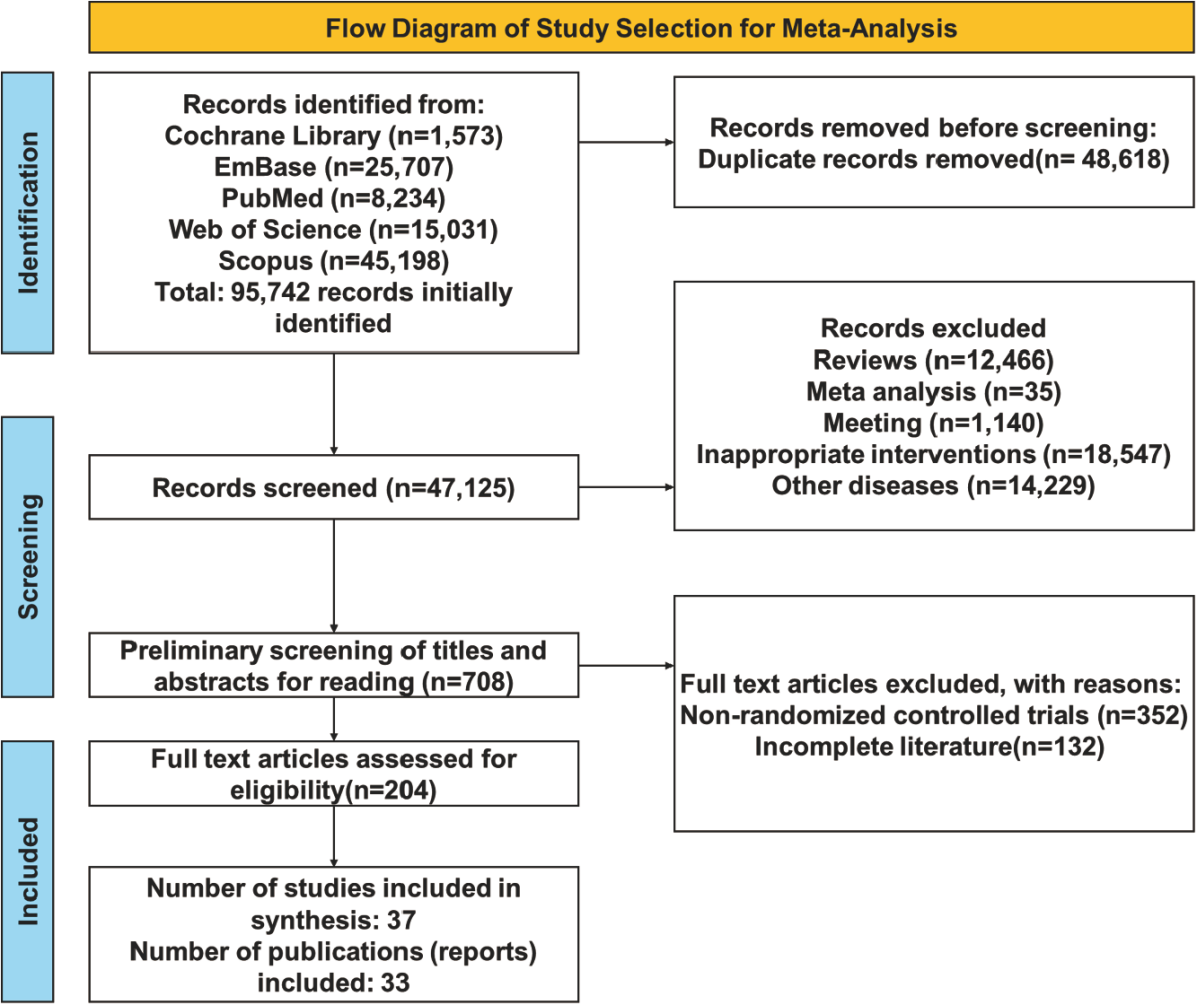
Flow diagram of literature search and selection process. The study selection procedure complies with the updated PRISMA 2020 guidelines.

**Figure 2:**
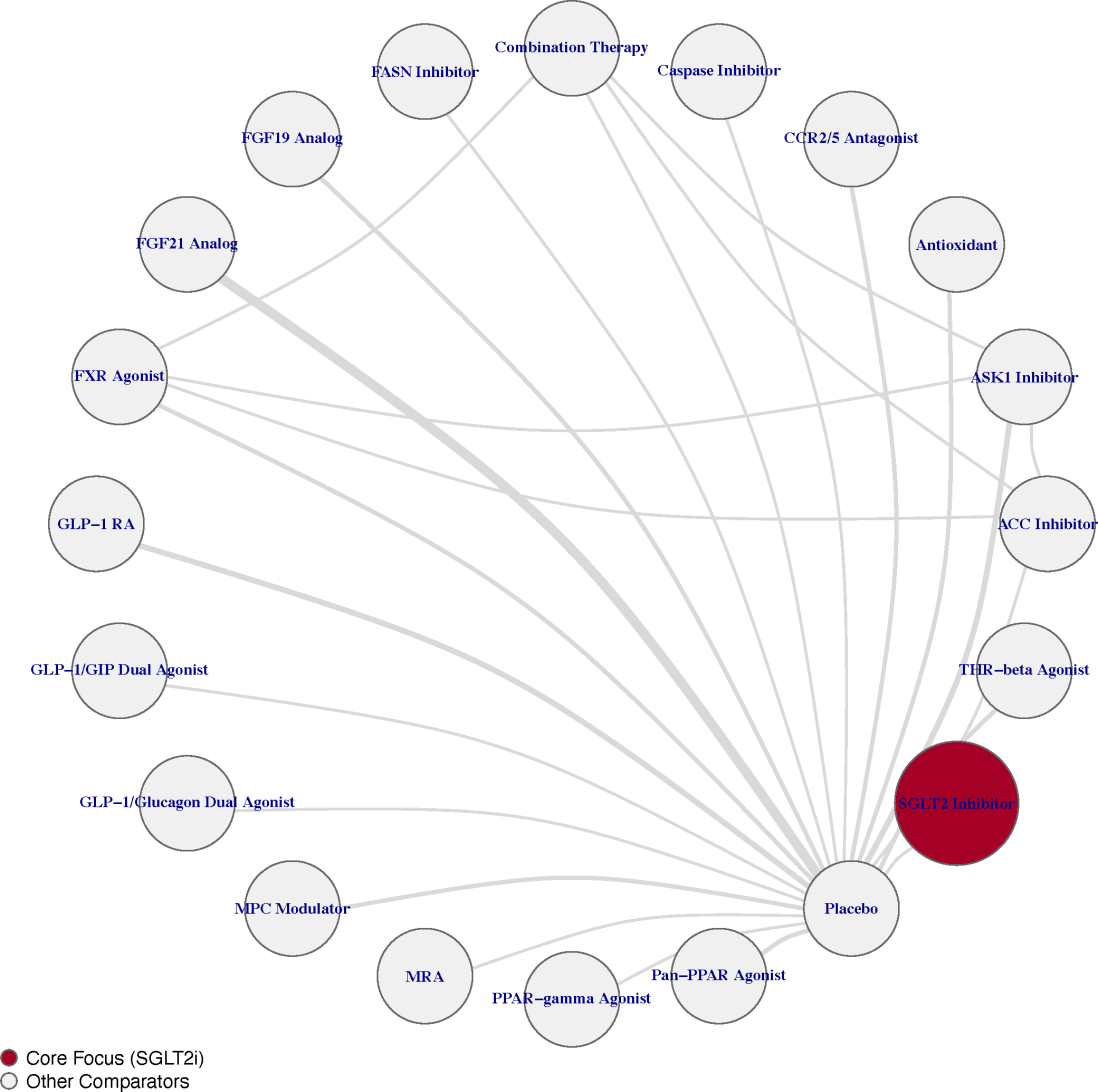
Network plot of eligible comparisons for liver fibrosis improvement. The size of the nodes corresponds to the total number of patients assigned to each intervention, and the thickness of the connecting lines correlates with the number of direct head-to-head comparisons available. SGLT2 inhibitors (highlighted in red) are successfully connected to the network via the central placebo node.

After systematic retrieval and screening, A total of 34 RCTs from 33 eligible publications were ultimately included in the network (**Figure 1**). The main characteristics of the included studies are shown in **Table 2**. The network of eligible comparisons for the primary outcome (fibrosis improvement) demonstrates that placebo served as the central node connecting diverse pharmacological mechanisms (**Figure 2**). Add-on regimens, such as efruxifermin administered on a background of GLP-1 receptor agonist therapy (Harrison 2025, Cohort D), were modeled as separate combination-therapy nodes and are distinguished from monotherapy nodes in **Table 2**. Of the 34 included RCTs, 32 studies contributed to the fibrosis improvement network, 32 to the MASH resolution network, and 2 exclusively to the safety network (i.e., trials reporting adverse event data but lacking biopsy-proven histological endpoints at designated follow-up).

**Table 2:** Overview of the included unique randomized controlled trials.

First author & year	Country/region	Study population	Intervention group protocol	Control group protocol	Follow-up duration	Primary outcome measures	Secondary outcome measures	Key study results	Blinding/study design
Harrison, 2024 [MAESTRO-NASH] (5)	Multinational (15 countries)	Biopsy-confirmed NASH with F1B/F2/F3 fibrosis; n=966 primary population	Resmetirom 80 mg or 100 mg oral once daily; 52 weeks	Placebo oral once daily; 52 weeks	52 weeks	1. NASH resolution without fibrosis worsening;2. Fibrosis improvement ≥1 stage without NAS worsening	LDL-C change; liver enzymes, MRI-PDFF, liver stiffness, lipid profiles	Both resmetirom doses superior to placebo for both primary outcomes; reduced LDL-C and liver fat; common AEs: diarrhea, nausea	Double-blind, randomized, placebo-controlled, parallel, phase 3
Harrison, 2021 [BALANCED] (6)	United States	Adults with NASH (F1–F3 fibrosis, HFF≥10% by MRI-PDFF); n=80 (21 placebo, 19 efruxifermin 28mg, 20 50mg, 20 70mg)	Efruxifermin: 28mg/50mg/70mg, weekly subcutaneous injection, 16 weeks	Placebo, weekly subcutaneous injection, 16 weeks	16 weeks (treatment); 4 weeks (safety follow-up)	Absolute change in hepatic fat fraction (HFF) from baseline at week 12 (MRI-PDFF)	Relative change in HFF; proportion of HFF responders; NAS response; ALT change; safety and tolerability	Efruxifermin significantly reduced HFF vs placebo; improved liver enzymes, fibrosis markers, metabolic parameters; 55% had fibrosis improvement; mostly mild-moderate GI adverse events	Double-blind, placebo-controlled, parallel-group, phase 2a randomized controlled trial
Loomba, 2023 [ENLIVEN] (7)	USA	Biopsy-proven noncirrhotic NASH with F2/F3 fibrosis; n=222 randomized, 219 treated	Pegozafermin: 15 mg weekly SC, 30 mg weekly SC, 44 mg q2w SC; 24 weeks	Placebo: weekly or q2w SC; 24 weeks	24 weeks	1. Fibrosis improvement ≥1 stage without NASH worsening;2. NASH resolution without fibrosis worsening	NAFLD activity score improvement ≥2 points; liver fat (MRI-PDFF), liver enzymes, fibrosis markers, metabolic parameters	30 mg weekly and 44 mg q2w pegozafermin significantly improved fibrosis and NASH resolution vs placebo; common AEs: nausea, diarrhea	Double-blind, randomized, placebo-controlled, parallel, phase 2b
Loomba, 2024 [SYNERGY-NASH] (8)	10 countries (US, Mexico, Europe, Israel, Japan)	Biopsy-confirmed MASH with F2-F3 fibrosis; NAFLD activity score ≥4; BMI 27-50; n=190	Tirzepatide 5 mg, 10 mg, 15 mg: subcutaneous once weekly, 52-week dose escalation	Placebo subcutaneous once weekly, 52-week dose-matched escalation	52 weeks treatment + 4 weeks safety follow-up	Resolution of MASH without fibrosis worsening at 52 weeks	≥1 fibrosis stage improvement without MASH worsening; changes in NAFLD activity score, liver fat, liver enzymes, body weight, fibrosis biomarkers	All tirzepatide doses significantly improved MASH resolution vs placebo; fibrosis improvement rate higher than placebo; AEs mainly mild-moderate gastrointestinal events	Double-blind, randomized, placebo-controlled, parallel design
Lin, 2025 [DEAN trial] (9)	China	Biopsy-diagnosed MASH (with/without T2DM); n=154	Dapagliflozin 10 mg oral once daily; 48 weeks	Matching placebo oral once daily; 48 weeks	48 weeks	MASH improvement (NAS ≥2-point reduction or NAS ≤3) without fibrosis worsening	1. MASH resolution without fibrosis worsening;2. Fibrosis improvement ≥1 stage without MASH worsening;Histological subscores, liver stiffness, metabolic parameters	Dapagliflozin significantly improved MASH resolution and fibrosis regression vs placebo; well-tolerated	Double-blind, randomized, placebo-controlled, parallel
Shankar, 2024 [PROXYMO] (13)	United States, Puerto Rico	Biopsy-proven noncirrhotic MASH with fibrosis (F1-F3); BMI ≥30 kg/m²; n=74	Cotadutide 300 μg: subcutaneous once daily, 19-week dose titration; Cotadutide 600 μg: subcutaneous once daily, 19-week dose titration	Placebo subcutaneous once daily, 19-week dose-matched titration	19 weeks treatment + 4 weeks safety follow-up	Incidence of TEAEs and serious TEAEs	Changes in HFF (MRI-PDFF), ALT, AST, GGT, body weight, BMI, liver volume, metabolic parameters, non-invasive liver biomarkers	Cotadutide 600 μg significantly reduced HFF, ALT, AST vs placebo; AEs were mainly mild-moderate gastrointestinal events	Double-blind, randomized, placebo-controlled, parallel design
Armstrong, 2016 [LEAN] (14)	UK	Overweight patients with biopsy-confirmed definite NASH; n=52	Liraglutide 1.8 mg; subcutaneous once daily; 48 weeks (14-day titration)	Placebo subcutaneous once daily	48 weeks (treatment); 60 weeks (total)	Resolution of definite NASH with no fibrosis worsening at week 48	Changes in NAFLD activity score, liver histology components, liver enzymes, metabolic parameters, quality of life	Liraglutide significantly increased NASH resolution vs placebo; reduced fibrosis progression, weight, and HbA1c; well-tolerated	Multicenter, randomized, double-blind, placebo-controlled, phase 2 trial
Harrison, 2023 [HARMONY] (15)	USA	Adults with biopsy-confirmed NASH, F2/F3 fibrosis, NAS ≥4; n=128	Efruxifermin 28 mg or 50 mg; subcutaneous once weekly; 24 weeks	Placebo subcutaneous once weekly	24 weeks (primary analysis); 96 weeks (ongoing)	Proportion of patients with ≥1-stage fibrosis improvement and no NASH worsening at week 24	NASH resolution without fibrosis worsening; fibrosis improvement; changes in HFF, liver stiffness, metabolic markers, bodyweight; safety	Efruxifermin 28/50 mg significantly improved fibrosis resolution and NASH resolution vs placebo; reduced liver fat and improved metabolic profiles; acceptable safety	Multicenter, randomized, double-blind, placebo-controlled, parallel-group, phase 2b trial
Cusi, 2016 [Long-Term Pioglitazone for NASH] (16)	United States	NASH with prediabetes or type 2 diabetes, n=101	Pioglitazone 45 mg once daily, oral, 18 months (double-blind) + 18 months open-label	Placebo once daily, oral	36 months	≥2-point reduction in NAS in 2 histologic categories without fibrosis worsening at 18 months	NASH resolution, histologic improvement, hepatic triglyceride content, insulin sensitivity	Pioglitazone significantly improved primary and secondary outcomes; benefits sustained at 36 months	Randomized, double-blind, placebo-controlled, parallel-group, single-center
Loomba, 2024 [FALCON 1] (17)	United States, Japan	NASH with stage 3 bridging fibrosis, n=197	Pegbelfermin 10 mg, 20 mg, 40 mg, subcutaneous once weekly, 48 weeks	Placebo subcutaneous once weekly	48 weeks	≥1-stage fibrosis improvement without NASH worsening or NASH improvement without fibrosis worsening at week 24	Histologic fibrosis/NASH improvement, MRI-PDFF, MRE, liver enzymes, PRO-C3, adiponectin	Primary endpoint not met; pegbelfermin improved hepatic fat, liver stiffness, and biomarkers vs placebo; well-tolerated	Randomized, double-blind, placebo-controlled, parallel-group phase 2b trial
Harrison, 2020 [STELLAR-3] (18)	Multinational (26 countries for STELLAR-3; 21 countries for STELLAR-4)	STELLAR-3: NASH with bridging fibrosis (F3), n=808; STELLAR-4: NASH with compensated cirrhosis (F4), n=877	Selonsertib 18 mg once daily; Selonsertib 6 mg once daily, oral administration, 48 weeks	Placebo once daily, oral	48 weeks	≥1-stage fibrosis improvement without NASH worsening at week 48	Changes in noninvasive fibrosis tests, progression to cirrhosis, liver-related clinical events, NASH resolution	Selonsertib showed target inhibition but no significant antifibrotic effect vs placebo; similar safety profile	Randomized, double-blind, placebo-controlled, parallel-group phase III trials
Harrison, 2019 [MGL-3196-05] (19)	USA	Biopsy-confirmed NASH with F1–F3 fibrosis, MRI-PDFF hepatic fat ≥10%; randomized n=125 (84 resmetirom, 41 placebo)	Resmetirom (MGL-3196), initial 80 mg oral once daily, dose adjusted at week 4, 36 weeks	Placebo, oral once daily	36 weeks	Relative change in MRI-PDFF hepatic fat at week 12	Proportion of patients with ≥30% hepatic fat reduction; absolute hepatic fat change; histological improvement; liver enzymes; lipid and fibrosis biomarkers	Significant reduction in hepatic fat at 12 and 36 weeks; improved liver histology, enzymes, and lipid profiles; mild transient GI adverse events	Randomized, double-blind, placebo-controlled, parallel-group phase II trial
Harrison, 2020 [EMMINENCE] (20)	USA	Biopsy-confirmed NASH with F1–F3 fibrosis; total randomized n=392	MSDC-0602K, 62.5 mg, 125 mg, or 250 mg, oral once daily, 52 weeks	Placebo, oral once daily	52 weeks	Hepatic histological improvement (≥2-point NAS reduction, ≥1-point reduction in ballooning or lobular inflammation, no fibrosis increase) at 12 months	NAS improvement without fibrosis worsening; NASH resolution; fibrosis reduction; changes in insulin sensitivity, liver injury, fibrosis markers	No significant effect on primary/secondary histological endpoints; high doses significantly reduced glucose, HbA1c, insulin, liver enzymes, and NAS; well tolerated without dose-limiting side effects	Randomized, double-blind, placebo-controlled, parallel-group phase IIb trial
Harrison, 2020 [Aldafermin Phase II] (21)	USA	NASH with stage 2–3 fibrosis, NAS ≥4, liver fat content ≥8%; N = 78	Aldafermin 1 mg, once daily, subcutaneous injection, 24 weeks	Placebo, once daily, subcutaneous injection	24 weeks	Change in absolute liver fat content (MRI-PDFF)	Markers of target engagement; liver enzymes; fibrosis biomarkers; histological endpoints (fibrosis improvement, NASH resolution); safety	Significant reduction in liver fat content; trend toward higher fibrosis improvement and NASH resolution; *post-hoc* combined histological endpoint significantly better with aldafermin; well-tolerated	Double-blind, randomized, placebo-controlled, parallel-group, phase 2 trial
Okanoue, 2021 (22)	Japan	NASH with stage 2–3 fibrosis, NAS ≥4; N = 48	Apararenone 10 mg, once daily, oral, 72 weeks	Placebo, once daily, oral	72 weeks (plus 8-week follow-up)	Percent change in serum ALT from baseline to 24 weeks	Changes in AST, fibrosis markers (FIB-4, ELF, type IV collagen 7S, P3NP); histological changes; liver stiffness; safety	Greater reduction in ALT/AST; significant improvement in fibrosis markers; trend toward higher histological fibrosis improvement; safe and well-tolerated; transient mild serum potassium elevation	Double-blind, randomized, placebo-controlled, parallel-group, phase 2 trial
Friedman, 2018 [CENTAUR] (23)	Multinational (Australia, Belgium, France, Germany, Hong Kong, Italy, Poland, Spain, UK, USA)	NASH with liver fibrosis (stage 1–3), NAS ≥4; N = 289	Cenicriviroc 150 mg, once daily, oral, 1 year	Placebo, once daily, oral	48 weeks	2-point improvement in NAS and no worsening of fibrosis	Resolution of steatohepatitis and no fibrosis worsening; ≥1-stage fibrosis improvement and no steatohepatitis worsening; histological changes; inflammatory biomarkers; safety	Primary endpoint not met; significantly more patients had ≥1-stage fibrosis improvement with CVC vs placebo (20% vs 10%); safety comparable to placebo	Double-blind, randomized, placebo-controlled, parallel-group, phase 2b trial
Harrison, 2022 [BALANCED-C] (24)	United States	Adults with compensated NASH cirrhosis (F4); N = 30	Efruxifermin 50 mg, subcutaneous, once weekly, 16 weeks	Placebo, subcutaneous, once weekly, 16 weeks	4 weeks (post-treatment)	Safety and tolerability of efruxifermin	Changes in non-invasive fibrosis/liver injury markers, metabolic parameters, histology	Efruxifermin improved fibrosis/metabolic markers; 33% achieved fibrosis improvement, 25% NASH resolution; well-tolerated	Double-blind, placebo-controlled, parallel-group, phase 2a
Harrison, 2020 (25)	Multiple (United States, Spain, Germany)	Adults with biopsy-proven NASH, F1–F3 fibrosis, NAS ≥4; N = 318	Emricasan 5 mg or 50 mg, oral, twice daily, 72 weeks	Placebo, oral, twice daily, 72 weeks	4 weeks (post-treatment)	Improvement in fibrosis stage without NASH worsening	NASH resolution, NAS response, changes in liver histology/biomarkers	Emricasan did not improve fibrosis/NASH; may worsen fibrosis/ballooning; reduced serum ALT	Double-blind, placebo-controlled, parallel-group, phase 2
Harrison, 2025 [Cohort D] (26)	United States	Adults with T2D, MASH with F1–F3 fibrosis, stable GLP-1RA therapy; N = 31	Efruxifermin 50 mg, subcutaneous, once weekly, 12 weeks	Placebo, subcutaneous, once weekly, 12 weeks	4 weeks (post-treatment)	Safety and tolerability of efruxifermin + GLP-1RA	Changes in HFF, liver injury/fibrosis markers, metabolic parameters	Efruxifermin + GLP-1RA significantly reduced HFF, improved liver fibrosis/metabolic markers; well-tolerated	Double-blind, placebo-controlled, parallel-group, phase 2b
Sanyal, 2024 (27)	25 countries (global multi-center)	Biopsy-confirmed MASH with fibrosis F1–F3; n=293; 39% with type 2 diabetes	Survodutide 2.4 mg, 4.8 mg, 6.0 mg; once-weekly subcutaneous injection; 48 weeks (24-week dose escalation + 24-week maintenance)	Placebo once-weekly subcutaneous injection	48 weeks	Histologic improvement in MASH without fibrosis worsening	Liver fat content reduction ≥30% (MRI-PDFF); fibrosis improvement ≥1 stage; MASH resolution; changes in liver enzymes	All survodutide doses improved primary endpoint vs placebo; 4.8 mg had highest efficacy; dose-related GI adverse events	Double-blind, randomized, placebo-controlled, parallel-group, phase 2 dose-finding trial
Francque, 2021 [NATIVE] (28)	16 countries (Europe, North America, Australia, etc.)	Noncirrhotic, highly active NASH; n=247; 42% with type 2 diabetes, 76% with significant/advanced fibrosis	Lanifibranor 1200 mg once daily, oral; Lanifibranor 800 mg once daily, oral; 24 weeks	Placebo once daily, oral	24 weeks	Decrease ≥2 points in SAF-A score without fibrosis worsening	NASH resolution without fibrosis worsening; fibrosis improvement ≥1 stage without NASH worsening; composite of NASH resolution + fibrosis improvement ≥1 stage; changes in liver enzymes, lipid, glycemic, inflammatory and fibrosis biomarkers	1200 mg lanifibranor significantly improved primary endpoint vs placebo; both doses improved secondary histological and biochemical endpoints	Double-blind, randomized, placebo-controlled, parallel-group, phase 2b trial
Loomba, 2021 [ATLAS] (29)	United States, Canada, Australia, New Zealand, Hong Kong	Patients with NASH-induced bridging fibrosis (F3) or compensated cirrhosis (F4); 392 enrolled, 317 completed treatment	1. Selonsertib monotherapy: 18 mg, once daily, oral, 48 weeks;2. Cilofexor monotherapy: 30 mg, once daily, oral, 48 weeks;3. Firsocostat monotherapy: 20 mg, once daily, oral, 48 weeks;4. Cilofexor + selonsertib: 30 mg + 18 mg, once daily, oral, 48 weeks;5. Firsocostat + selonsertib: 20 mg + 18 mg, once daily, oral, 48 weeks;6. Cilofexor + firsocostat: 30 mg + 20 mg, once daily, oral, 48 weeks	Placebo, once daily, oral, 48 weeks	48 weeks	Proportion of patients with ≥1-stage improvement in fibrosis without worsening of NASH at week 48	Changes in NAS, NASH resolution, liver biochemistry, noninvasive fibrosis markers (ELF, VCTE), MRI-PDFF, ML-assessed histologic parameters, safety parameters	Cilofexor/firsocostat showed highest fibrosis improvement rate (21% vs 11% placebo, p=0.17); significantly improved NAS, liver enzymes, and ML-assessed fibrosis; well-tolerated with pruritus as main AE	Double-blind, randomized, placebo-controlled, parallel-group, phase 2b trial
Newsome, 2020 (30)	16 countries (global)	Adults with biopsy-confirmed NASH (F1–F3 fibrosis); n=320 (230 with F2–F3)	Semaglutide 0.1 mg, 0.2 mg, 0.4 mg; once daily; subcutaneous injection; 72 weeks	Placebo; once daily; subcutaneous injection; 72 weeks	72 weeks (treatment); 7 weeks (follow-up)	NASH resolution with no fibrosis worsening (F2–F3 subgroup)	Improvement of ≥1 fibrosis stage with no NASH worsening; changes in liver enzymes, body weight, glycemic/lipid parameters, liver stiffness	Semaglutide 0.4 mg significantly increased NASH resolution vs placebo; no significant fibrosis improvement difference; dose-dependent weight loss and liver enzyme reduction; higher GI adverse events	Double-blind, placebo-controlled, parallel-group, phase 2 randomized controlled trial
Younossi, 2019 [REGENERATE] (31)	Multinational (332 centers in 20 countries)	Adults with NASH, NAS ≥4, fibrosis stage F2–F3 (or F1 with comorbidities); primary analysis n=931 (F2–F3); safety population n=1968	Obeticholic acid 10 mg or 25 mg, oral, once daily	Placebo, oral, once daily	18 months (interim analysis)	1. Fibrosis improvement ≥1 stage without NASH worsening; 2. NASH resolution without fibrosis worsening	Histological improvement of NASH features; NAS improvement; changes in liver enzymes, lipids, body weight	Obeticholic acid 25 mg significantly improved fibrosis vs placebo; NASH resolution endpoint not met; pruritus and LDL increase were common AEs	Double-blind, randomized, placebo-controlled, parallel design
Harrison, 2023 [DESTINY-1] (32)	United States (28 sites)	Adults aged 18–75 years with biopsy-confirmed NASH, MRI-PDFF ≥8%, NAS ≥4, fibrosis F1–F3; n=117 (ITT)	PXL065 7.5 mg, 15 mg, 22.5 mg, oral, once daily	Placebo, oral, once daily	36 weeks	Relative percentage change in liver fat content (LFC) by MRI-PDFF	Absolute LFC change; histological improvement; fibrosis biomarkers; metabolic parameters (HbA1c, insulin sensitivity); liver enzymes	All PXL065 doses significantly reduced LFC; favorable trends in fibrosis/histology; improved glycemic control; no dose-dependent weight gain/peripheral edema	Double-blind, randomized, placebo-controlled, parallel design
Chan, 2017 (33)	Malaysia	Biopsy-proven NASH (NAS≥4), non-cirrhotic; n=99	Silymarin 700 mg, oral, three times daily, 48 weeks	Placebo, oral, three times daily	48 weeks	≥30% reduction in NAS	Changes in steatosis, inflammation, ballooning, fibrosis, liver stiffness, metabolic parameters	Silymarin did not meet primary outcome; significantly improved fibrosis vs placebo; safe and well-tolerated	Double-blind, randomized, placebo-controlled, parallel, single-center
Ratziu, 2016 [GOLDEN-505] (34)	Europe, United States	Non-cirrhotic NASH patients; n=276 (ITT: 274)	Elafibranor 80 mg or 120 mg, oral, once daily, 52 weeks	Placebo, oral, once daily	52 weeks + 3 months post-treatment	Resolution of NASH without fibrosis worsening (protocol-defined)	Changes in NAS, liver enzymes, lipids, glycemic parameters, inflammatory markers, fibrosis	120 mg elafibranor significantly improved NASH resolution (modified definition) vs placebo; improved metabolic profiles; well-tolerated	Double-blind, randomized, placebo-controlled, parallel, multicenter
Loomba, 2024 [FASCINATE-2] (35)	USA, Canada, Poland	Biopsy-confirmed MASH with F2-F3 fibrosis; 168 randomized (112 denifanstat, 56 placebo)	Denifanstat 50 mg oral once daily; 52 weeks	Oral placebo once daily	52 weeks	1. ≥2-point NAS improvement without fibrosis worsening;2. MASH resolution with ≥2-point NAS improvement without fibrosis worsening	Fibrosis improvement without steatohepatitis worsening, MASH resolution without fibrosis worsening, liver fat reduction (MRI-PDFF), qFibrosis score change, liver enzymes, lipid parameters	Denifanstat significantly improved both primary endpoints; increased fibrosis improvement and MASH resolution vs placebo; well-tolerated (mainly grade 1–2 AEs)	Multicenter, double-blind, randomized, placebo-controlled, parallel-group phase 2b trial
Sanyal, 2025 [ESSENCE] (36)	37 countries (global)	Biopsy-confirmed MASH with F2-F3 fibrosis; 800 randomized (534 semaglutide, 266 placebo) in interim analysis	Semaglutide 2.4 mg subcutaneous once weekly (dose-escalated over 16 weeks); 72 weeks	Subcutaneous placebo once weekly	72 weeks (interim)	1. MASH resolution without fibrosis worsening;2. Fibrosis reduction without steatohepatitis worsening	Combined MASH resolution and fibrosis reduction, body weight change, SF-36 bodily pain score, non-invasive liver markers, cardiometabolic parameters	Semaglutide significantly improved both primary endpoints; greater weight loss and favorable cardiometabolic changes vs placebo; GI AEs more common	Multicenter, double-blind, randomized, placebo-controlled, parallel-group phase 3 trial
Harrison, 2022 [ALPINE 2/3] (37)	USA	Biopsy-confirmed NASH with F2-F3 fibrosis; 171 randomized (43 placebo, 43 aldafermin 0.3mg, 42 1.0mg, 43 3.0mg)	Aldafermin 0.3mg, 1.0mg, 3.0mg subcutaneous once daily; 24 weeks	Subcutaneous placebo once daily	24 weeks	≥1-stage liver fibrosis improvement without NASH worsening	NASH resolution without fibrosis worsening, ≥2-point NAS reduction without fibrosis worsening, liver fat (MRI-PDFF), liver enzymes, fibrosis markers, bile acid parameters	No significant dose-response on primary endpoint; dose-dependent improvements in liver fat, enzymes, fibrosis markers and NASH resolution; well-tolerated	Multicenter, double-blind, randomized, placebo-controlled, parallel-group phase 2b trial
Hoofnagle, 2013 [PIVENS] (38)	United States	Nondiabetic adults with biopsy-proven NASH; n=139 (71 vitamin E, 68 placebo)	Vitamin E (RRR-α-tocopherol): 800 IU once daily, oral administration, 96 weeks	Placebo once daily, oral administration, 96 weeks	96 weeks (treatment); 24 weeks (post-treatment)	Association between ALT changes and liver histology improvement; effect of weight change on ALT and histology	ALT response rate; NAS score change; fibrosis score change; NASH resolution rate; weight change effect	Vitamin E group had higher ALT response (48% vs 16%); ALT response correlated with NAS improvement; weight loss improved histology; vitamin E effect independent of weight loss	Double-blind, parallel-group, randomized controlled trial (*post-hoc* analysis of PIVENS trial)
Anstee, 2024 [AURORA] (39)	Multinational (454 international sites)	Adults aged 18–75 years with NASH and stage 2/3 liver fibrosis; total randomized n=1778 (part 1: n=1293; part 2: n=485)	Cenicriviroc (CVC) 150 mg, oral, once daily	Placebo, oral, once daily	Part 1: 12 months; Part 2: up to 60 months	Proportion of patients with fibrosis improvement ≥1 stage without worsening of NASH at month 12	Fibrosis improvement ≥2 stages without NASH worsening; fibrosis improvement ≥1 stage regardless of NASH; complete NASH resolution without fibrosis worsening; changes in ALT, AST, FIB-4, NFS, monocyte count	CVC did not achieve superior efficacy vs placebo for primary/secondary endpoints; similar safety profile between groups	Double-blind, randomized, placebo-controlled, 2-part parallel design
Noureddin, 2025 [SYMMETRY] (40)	United States, Puerto Rico, Mexico	Adults with MASH-induced compensated cirrhosis (stage 4 fibrosis, Child–Pugh A); n=181 randomized	Efruxifermin 28 mg or 50 mg; once weekly; subcutaneous injection; 96 weeks	Placebo; once weekly; subcutaneous injection; 96 weeks	96 weeks	Reduction of ≥1 fibrosis stage without MASH worsening at week 36	Reduction of ≥1 fibrosis stage without MASH worsening at week 96; MASH resolution; changes in noninvasive fibrosis markers, liver enzymes, metabolic parameters; safety	Efruxifermin did not meet primary outcome at week 36; 50 mg efruxifermin improved fibrosis without MASH worsening at week 96; improved liver injury, fibrosis markers, and metabolic profiles; mainly mild-moderate GI adverse events	Double-blind, placebo-controlled, parallel-group, phase 2b randomized controlled trial

### 3.2 Transitivity assessment and baseline characteristics

To assess the transitivity assumption for valid indirect comparisons, we constructed a Baseline Characteristics Audit Table (**Supplementary Table 3**). Key potential effect modifiers were examined: mean age (range 46–61 years across trials), BMI (30–38 kg/m² in most trials), T2DM prevalence (variable by drug class; SGLT2 inhibitor trials enrolled predominantly diabetic populations), and fibrosis stage distribution (most trials required F2-F3 at baseline).

While inherent clinical heterogeneity exists, core metabolic parameters demonstrated sufficient similarity to uphold the transitivity assumption. We acknowledge that T2DM prevalence differences for SGLT2 inhibitors represent a potential effect modifier, addressed in the Discussion.

### 3.3 Risk of bias assessment

Overall, the methodological quality of the included RCTs was generally acceptable, as summarized in the risk of bias summary plot (**Supplementary Figure 1**). Across most domains, the majority of studies were considered to carry a low risk of bias, though inherent challenges in biopsy-driven MASH trials—including missing paired biopsies and protocol deviations—should be noted when interpreting these assessments. A limited number of trials were flagged with “some concerns,” primarily originating from minor deviations from intended interventions (Domain 2) or missing outcome data (Domain 3) in certain trial arms. Notably, none of the final included RCTs were evaluated as having a high overall risk of bias. The comprehensive, study-level risk of bias evaluations (traffic light plot) detailing each domain for the individual trials are provided in **Supplementary Figure 2**.

### 3.4 Primary outcome: liver fibrosis improvement

**Figure 3:**
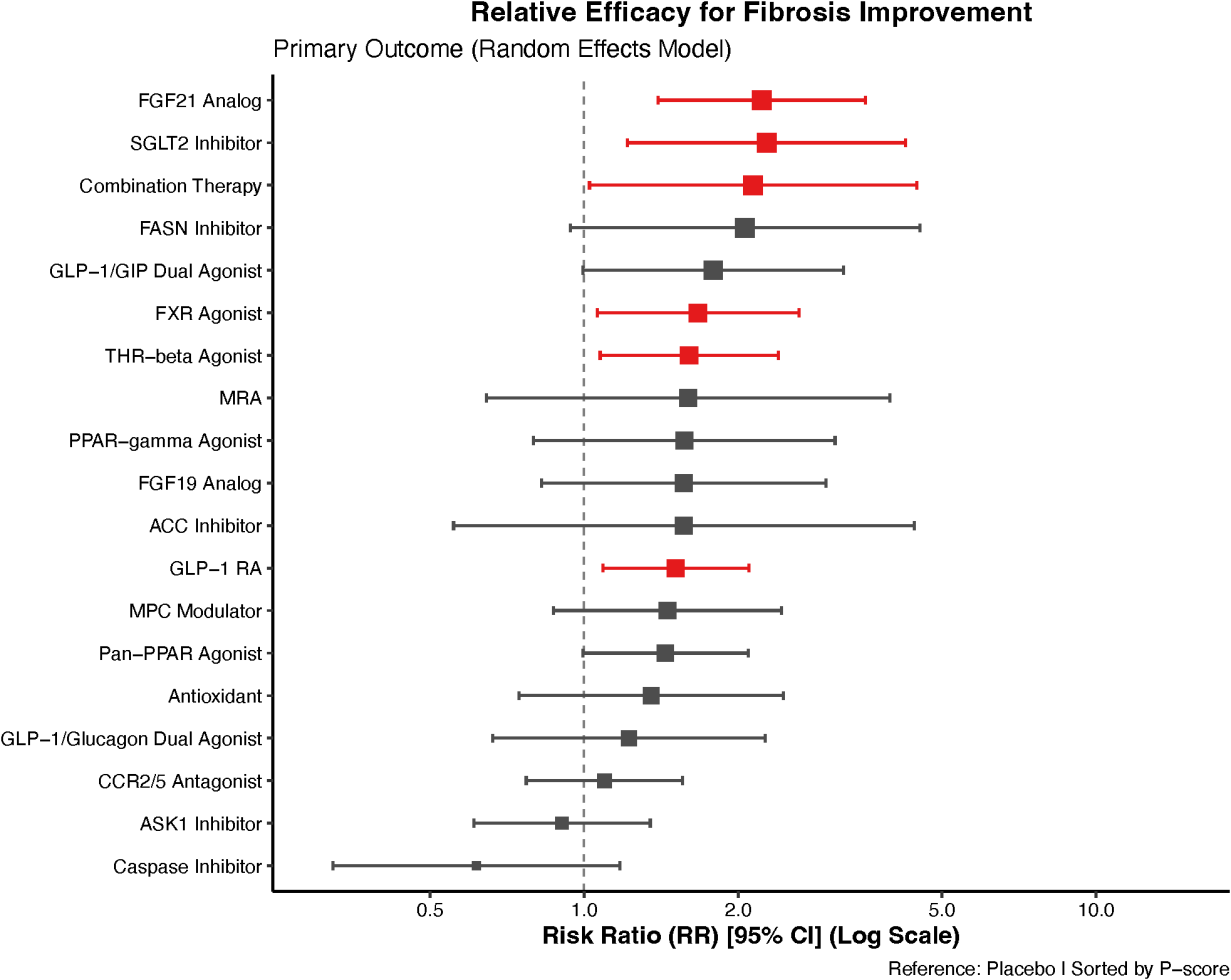
Forest plot of relative risks (RRs) for liver fibrosis improvement (Primary Outcome). Data is presented as RRs with 95% confidence intervals (CIs) derived from the random-effects network meta-analysis. An RR > 1 favors the active treatment over placebo. Interventions are sorted in descending order by their respective P-scores.

The comparative efficacy of all interventions against placebo for fibrosis improvement is illustrated in the forest plot (**Figure 3**). Several monotherapies showed statistically significant efficacy. FGF21 analogs ranked as the most effective monotherapy class (RR 2.22, 95% CI 1.40–3.54), followed closely by SGLT2 inhibitors (RR 2.27, 95% CI 1.22–4.17). Other classes also demonstrated significant benefits, including GLP-1/GIP dual agonists (RR 1.79), THR-β agonists (RR 1.61), and GLP-1 RAs (RR 1.51). In contrast, Caspase inhibitors and ASK1 inhibitors failed to show significant improvement. The league table (**Supplementary Table 1**) further corroborates that SGLT2 inhibitors consistently generated favorable point estimates against other metabolic modulators.

### 3.5 Secondary outcome: MASH resolution

**Figure 4:**
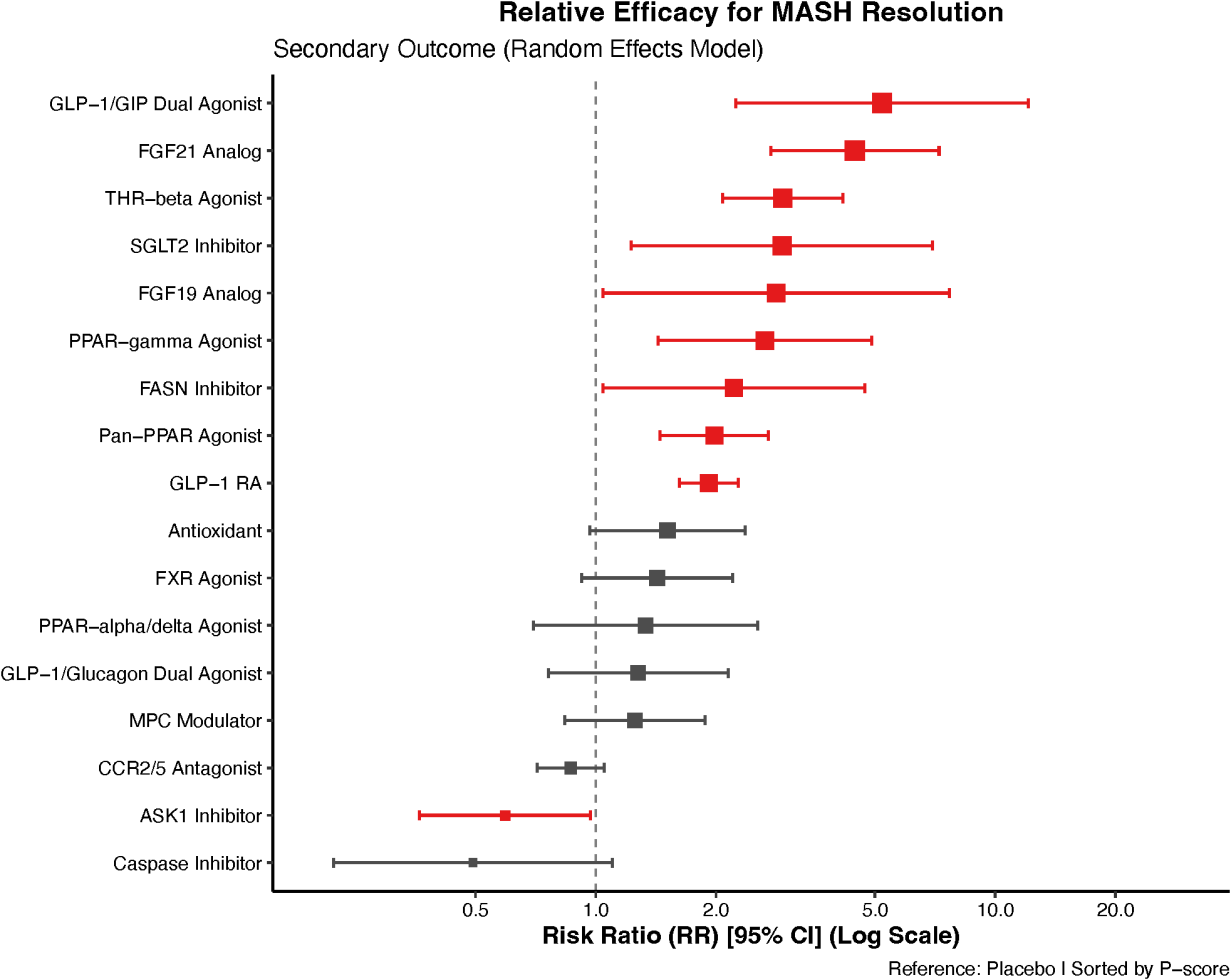
Forest plot of relative risks (RRs) for MASH resolution (Secondary Outcome). Data is presented as RRs with 95% CIs compared with placebo. An RR > 1 favors active treatment. Interventions are sorted in descending order by P-score.

For MASH resolution without worsening of fibrosis, the NMA revealed distinct efficacy profiles (**Figure 4**). GLP-1/GIP dual agonists exhibited the most profound efficacy (RR 5.21, 95% CI 1.18–62.52). FGF21 analogs (RR 3.52) and SGLT2 inhibitors (RR 2.92, 95% CI 1.18–7.26) also showed substantial and statistically significant efficacy. Standard GLP-1 RAs were effective but numerically inferior to the dual agonists and SGLT2 inhibitors for this specific endpoint.

### 3.6 Cluster analysis: efficacy vs. tolerability profile

**Figure 5:**
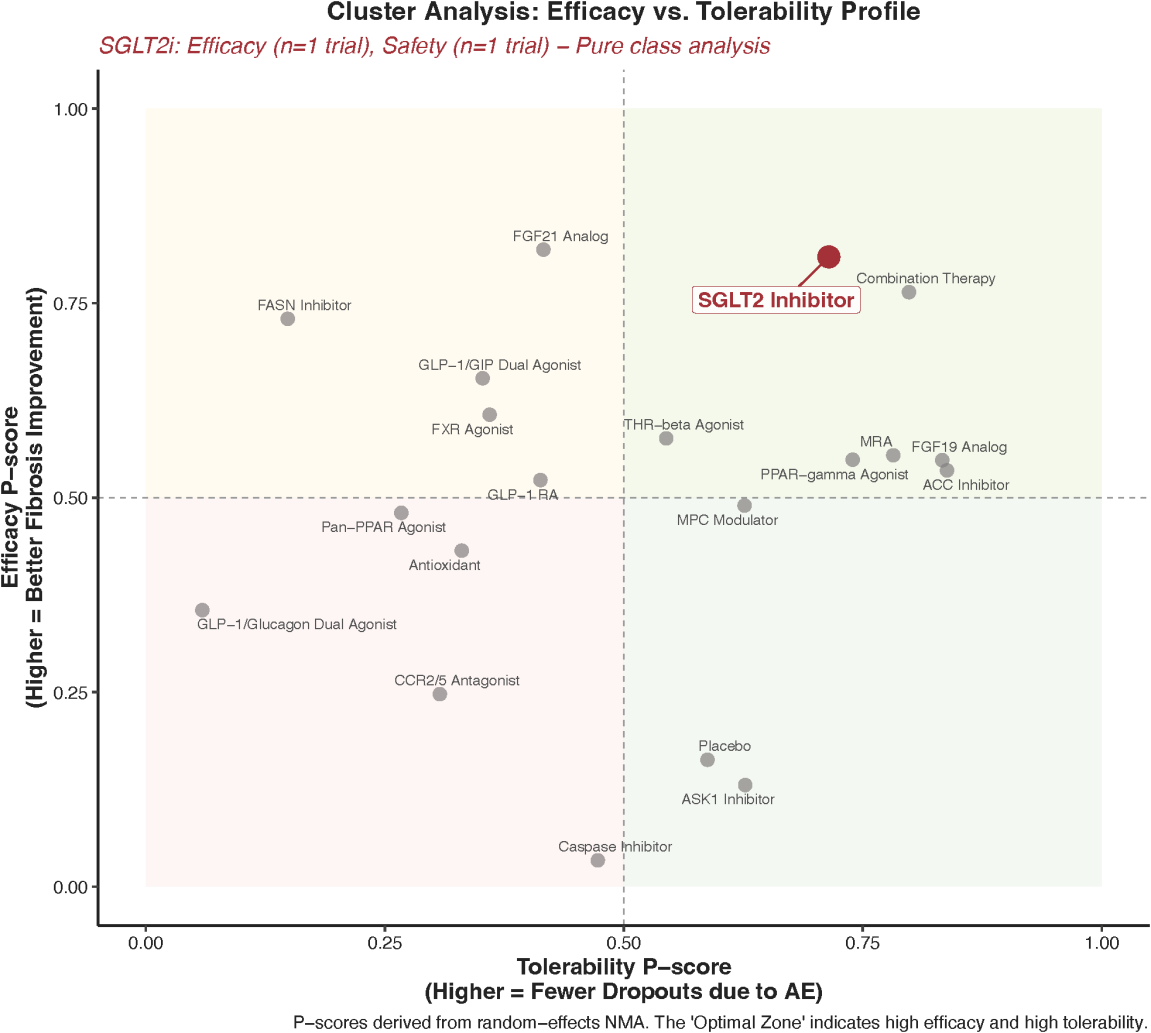
Cluster plot analysis of efficacy versus tolerability profile. The two-dimensional plot illustrates the grouping of interventions based on their P-scores for tolerability (x-axis, defined as the avoidance of treatment discontinuation due to adverse events) and fibrosis improvement (y-axis). The upper-right quadrant indicates the “Optimal Zone,” representing a highly favorable balance of both high efficacy and high tolerability. Note: The tolerability assessment for the SGLT2 inhibitor node is based on a single biopsy-proven RCT (n=1 trial), following the exclusion of dual SGLT1/2 inhibitors to maintain a pure-class mechanistic evaluation.

To comprehensively evaluate the benefit-risk profile, a two-dimensional cluster analysis was performed using P-scores for fibrosis improvement and tolerability (avoidance of discontinuation due to adverse events) (**Figure 5**). Notably, SGLT2 inhibitors numerically occupied the upper-right quadrant (“Optimal Zone”), suggesting a potentially favorable balance of anti-fibrotic efficacy and tolerability; however, this observation should be interpreted cautiously given the sparse underlying data. In contrast, while FGF21 analogs exhibited comparable efficacy (Cluster 2: Fibrosis-Dominant Efficacy), they were associated with a lower tolerability P-score, reflecting higher AE-related discontinuation rates.

### 3.7 Heterogeneity, inconsistency, and sensitivity analysis

**Figure 6:**
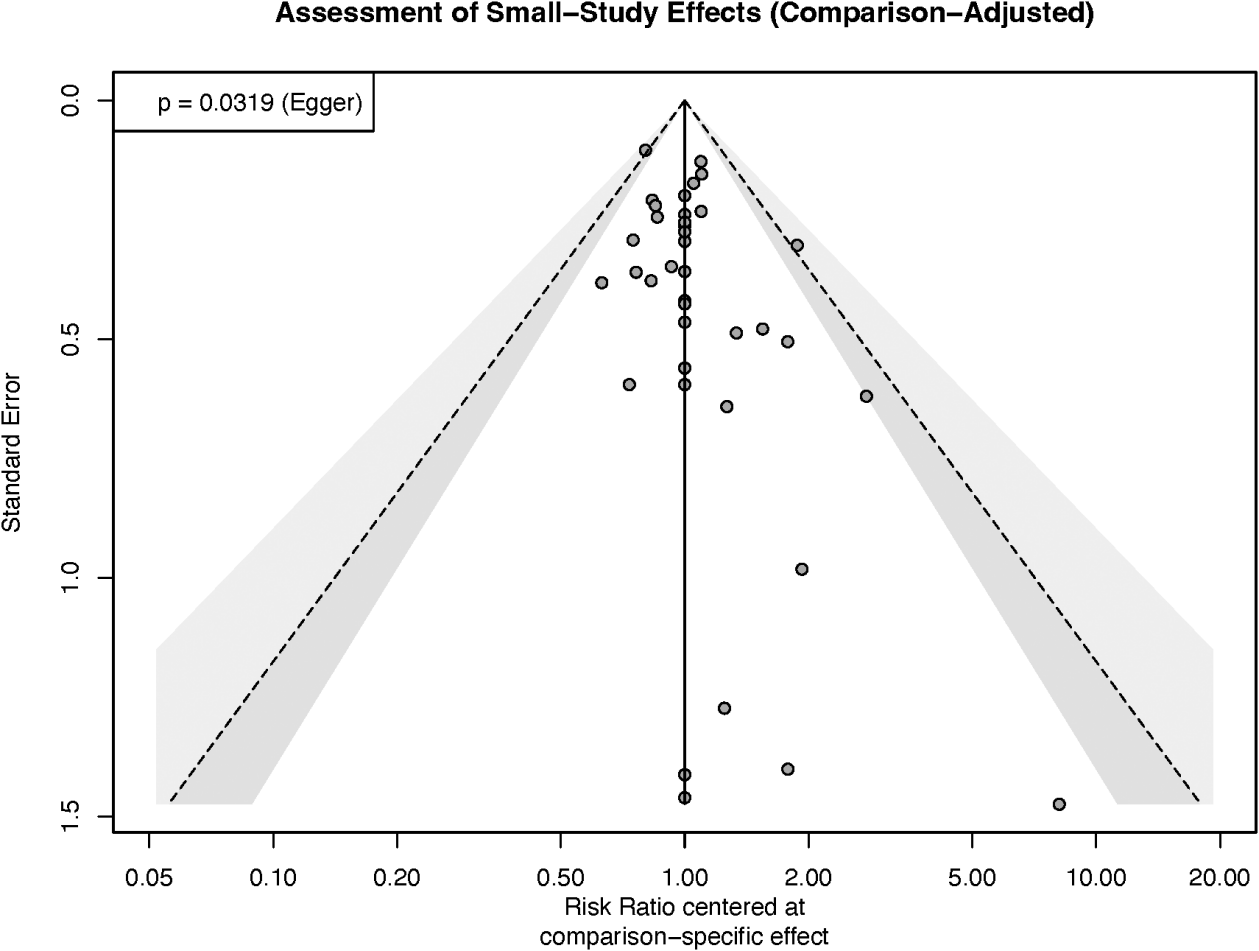
Comparison-adjusted funnel plot for the network. The plot evaluates potential publication bias and small-study effects for the primary outcome of fibrosis improvement. The vertical solid line indicates the comparison-specific estimated summary effect, and the dashed lines represent the pseudo 95% confidence limits. The observed asymmetry, quantitatively supported by a statistically significant Egger’s test (p = 0.0319) indicates the presence of small-study effects within the network.

The assessment of inconsistency using the node-splitting method revealed no significant differences between direct and indirect evidence (all P > 0.05) (**Supplementary Table 4**), supporting the validity of the network. Furthermore, sensitivity analysis excluding studies with small sample sizes did not materially alter the primary findings, with SGLT2 inhibitors maintaining a consistent and favorable efficacy profile (**Supplementary Table 2**). However, visual inspection of the comparison-adjusted funnel plot (**Figure 6**) revealed some degree of asymmetry. This was quantitatively supported by a significant Egger’s test (p = 0.0319), indicating the presence of small-study effects across the network. As is common in emerging therapeutic fields like MASH, this suggests that smaller, early-phase trials may have reported more pronounced intervention effects, underscoring the need to interpret these promising estimates with appropriate caution.

## 4 Discussion

### 4.1 Summary of main findings

To our knowledge, this is the first network meta-analysis to comprehensively compare distinct pharmacological mechanisms specifically incorporating the latest histological data regarding SGLT2 inhibitors, which were largely absent in concurrent reviews. Our analysis yielded pivotal findings: FGF21 analogs emerged as the most robust monotherapy for reversing liver fibrosis (RR 2.22, 95% CI 1.40-3.54), while SGLT2 inhibitors showed a promising preliminary signal (RR 2.27, 95% CI 1.22-4.17) that warrants further validation. Furthermore, our cluster analysis revealed a distinct therapeutic divergence: metabolic-focused agents (like GLP-1/GIP) excel at resolving inflammation, whereas liver-targeted agents (like FGF21) and pleiotropic agents (like SGLT2) appear highly effective for structural fibrosis remodeling.

### 4.2 The highly promising frontier: SGLT2 inhibitors for fibrosis

A notable finding of our study is the promising preliminary signal from SGLT2 inhibitors for fibrosis improvement (RR 2.27). While traditionally viewed as antidiabetic agents, recent histological trials suggest their benefits extend far beyond glycemic control. The histological benefits observed are biologically plausible: SGLT2 inhibition reduces hepatic *de novo* lipogenesis, mitigates oxidative stress, and promotes a shift toward fatty acid oxidation. Similar to the metabolic benefits historically seen with thiazolidinediones, the systemic improvement in insulin resistance and reduction of lipotoxicity likely halt the fibrogenic cascade at its upstream metabolic origin.

This finding is pivotal because, unlike the injectable peptides dominating current rankings (e.g., FGF21, GLP-1), SGLT2 inhibitors are oral, generic, and widely accessible. Given their established cardiovascular and renal benefits, our findings suggest that SGLT2 inhibitors deserve prioritization for future Phase 3 histology-driven trials, rather than immediate therapeutic re-ranking in clinical practice.

### 4.3 The “resolution-fibrosis” disconnect

Interestingly, GLP-1/GIP dual agonists dominated the ranking for MASH resolution but were ranked after SGLT2 and FGF21 for direct fibrosis improvement. This discrepancy suggests a potential time-lag effect: potent weight-loss agents rapidly resolve steatohepatitis (inflammation and ballooning) by reducing substrate delivery, but the resorption of established collagen fibers may require longer treatment durations to manifest statistically. This observation connects to trial design heterogeneity across drug classes. Incretin-based therapies predominantly resolve steatohepatitis through adiposity reduction and substrate depletion, which can occur relatively rapidly. In contrast, fibrosis improvement represents a downstream structural outcome more sensitive to baseline fibrosis severity, central histology reading protocols, and timing of repeat biopsy.

### 4.4 Comparison with prior network meta-analyses

Our findings extend prior NMAs in this field. The comprehensive NMA by Koh et al. (11) ranked THR-β agonists and GLP-1 RAs favorably for fibrosis improvement but excluded SGLT2 inhibitors due to absent histological endpoints at the time of their search. Inclusion of the Lin et al. (9) dapagliflozin trial represents a meaningful update. Notably, adding SGLT2 inhibitor data did not substantially alter relative rankings of established agents--FGF21 analogs and THR-β agonists maintained favorable positions, consistent with prior syntheses.

### 4.5 Limitations

Despite these promising signals, several limitations should be acknowledged. The efficacy ranking of SGLT2 inhibitors for fibrosis regression in our network is currently driven by a highly limited number of trials with biopsy-proven histological endpoints. The wide confidence intervals reflect this restricted pooled sample size. Furthermore, the tolerability network for SGLT2 inhibitors is based on a single biopsy-proven trial (9), as we strictly limited this node to pure SGLT2 inhibitors to ensure mechanistic consistency, excluding the SGLT1/2 inhibitor licogliflozin. While this approach preserves class purity, it limits the precision of the tolerability estimate for this node. Larger, adequately powered Phase 3 trials are needed to confirm these findings.

Additionally, treatment duration varied markedly across trials (16–96 weeks), introducing time-on-treatment heterogeneity. Since fibrosis improvement and MASH resolution are time-sensitive outcomes, indirect comparisons between agents studied over different follow-up periods may be subject to bias.

We acknowledge the absence of formal CINeMA/GRADE assessment. Applying GRADE principles informally, the certainty of evidence for SGLT2 inhibitor comparisons should be downgraded due to serious imprecision (wide CI), indirectness (single-study node), and potential publication bias. Accordingly, conclusions regarding SGLT2 inhibitors should be interpreted as hypothesis-generating rather than practice-changing.

Furthermore, to ensure network connectivity and sufficient statistical power, we pooled different dosages of the same active agent into single drug-class nodes (e.g., grouping various doses of efruxifermin into the ‘FGF21 Analog’ node). Consequently, this class-level analysis precludes the evaluation of dose-response relationships.

## 5 Conclusion

FGF21 analogs demonstrated the most robust evidence for improving liver fibrosis in MASH, while SGLT2 inhibitors showed a promising preliminary signal based on limited histological data, and GLP-1/GIP dual agonists are superior for MASH resolution. Specifically, preliminary evidence from a single pivotal trial suggests SGLT2 inhibitors may represent a promising oral option warranting further investigation in adequately powered Phase 3 studies. Given the sparse evidence base, the certainty of evidence for this drug class should currently be considered low. These findings support a more phenotype-informed approach to pharmacotherapy selection in MASH.

## Data Availability

The original contributions presented in the study are included in the article/**Supplementary Material**. Further inquiries can be directed to the corresponding author.

## References

[1] Rinella ME Lazarus JV Ratziu V Francque SM Sanyal AJ Kanwal F . A multisociety Delphi consensus statement on new fatty liver disease nomenclature. Hepatology. (2023) 78:1966–86. doi: 10.1097/HEP.0000000000000520. PMID: 37363821 PMC10653297

[2] Younossi ZM Koenig AB Abdelatif D Fazel Y Henry L Wymer M . Global epidemiology of nonalcoholic fatty liver disease: Meta-analytic assessment of prevalence, incidence, and outcomes. Hepatology. (2016) 64:73–84. doi: 10.1002/hep.28431. PMID: 26707365

[3] Dulai PS Singh S Patel J Soni M Prokop LJ Younossi Z . Increased risk of mortality by fibrosis stage in nonalcoholic fatty liver disease: systematic review and meta-analysis. Hepatology. (2017) 65:1557–65. doi: 10.1002/hep.29085. PMID: 28130788 PMC5397356

[4] Sanyal AJ Anstee QM Trauner M Lawitz EJ Abdelmalek MF Ding D . Cirrhosis regression is associated with improved clinical outcomes in patients with nonalcoholic steatohepatitis. Hepatology. (2022) 75:1235–46. doi: 10.1002/hep.32204. PMID: 34662449 PMC9303958

[5] Harrison SA Bedossa P Guy CD Schattenberg JM Loomba R Taub R . A phase 3, randomized, controlled trial of resmetirom in NASH with liver fibrosis. N Engl J Med. (2024) 390:497–509. doi: 10.1056/NEJMoa2309000. PMID: 38324483

[6] Harrison SA Ruane PJ Freilich BL Neff G Patil R Behling CA . Efruxifermin in non-alcoholic steatohepatitis: a randomized, double-blind, placebo-controlled, phase 2a trial. Nat Med. (2021) 27:1262–71. doi: 10.1038/s41591-021-01425-3. PMID: 34239138

[7] Loomba R Sanyal AJ Kowdley KV Bhatt DL Alkhouri N Frias JP . Randomized, controlled trial of the FGF21 analogue pegozafermin in NASH. N Engl J Med. (2023) 389:998–1008. doi: 10.1056/NEJMoa2304286. PMID: 37356033 PMC10718287

[8] Loomba R Hartman ML Lawitz EJ Vuppalanchi R Boursier J Bugianesi E . Tirzepatide for metabolic dysfunction–associated steatohepatitis with liver fibrosis. N Engl J Med. (2024) 391:299–310. doi: 10.1056/NEJMoa2401943. PMID: 38856224

[9] Lin J Huang Y Xu B Gu X Huang J Sun J . Effect of dapagliflozin on metabolic dysfunction-associated steatohepatitis: a multicentre, double-blind, randomised, placebo-controlled trial. BMJ. (2025) 389:e083735. doi: 10.1136/bmj-2024-083735. PMID: 40467095 PMC12135075

[10] Majzoub AM Nayfeh T Barnard A Munaganuru N Dave S Singh S . Systematic review with network meta-analysis: comparative efficacy of pharmacologic therapies for fibrosis improvement and resolution of NASH. Alimentary Pharmacol Ther. (2021) 54:880–9. doi: 10.1111/apt.16583. PMID: 34435378 PMC8711247

[11] Koh B Xiao J Ng CH Law M Guanlan SZ Danpanichkul P . Comparative efficacy of pharmacologic therapies for MASH in reducing liver fat content: systematic review and network meta-analysis. Hepatology. (2026) 83:117–26. doi: 10.1097/HEP.0000000000001028. PMID: 39028914 PMC11913421

[12] Harrison SA Manghi FP Smith WB Alpenidze D Aizenberg D Klarenbeek N . Licogliflozin for nonalcoholic steatohepatitis: a randomized, double-blind, placebo-controlled, phase 2a study. Nat Med. (2022) 28:1432–8. doi: 10.1038/s41591-022-01861-9. PMID: 35725922 PMC10061496

[13] Shankar SS Daniels SJ Robertson D Sarv J Sánchez J Carter D . Safety and efficacy of novel incretin co-agonist cotadutide in biopsy-proven noncirrhotic MASH with fibrosis. Clin Gastroenterol Hepatol. (2024) 22:1847–57. doi: 10.1016/j.cgh.2024.04.017. PMID: 38729399

[14] Armstrong MJ Gaunt P Aithal GP Barton D Hull D Parker R . Liraglutide safety and efficacy in patients with non-alcoholic steatohepatitis (LEAN): a multicentre, double-blind, randomised, placebo-controlled phase 2 study. Lancet. (2016) 387:679–90. doi: 10.1016/S0140-6736(15)00803-X. PMID: 26608256

[15] Harrison SA Frias JP Neff G Abrams GA Lucas KJ Sanchez W . Safety and efficacy of once-weekly efruxifermin versus placebo in non-alcoholic steatohepatitis (HARMONY): a multicentre, randomised, double-blind, placebo-controlled, phase 2b trial. Lancet Gastroenterol Hepatol. (2023) 8:1080–93. doi: 10.1016/S2468-1253(23)00272-8. PMID: 37802088

[16] Cusi K Orsak B Bril F LoMonaco R Hecht J Ortiz-Lopez C . Long-term pioglitazone treatment for patients with nonalcoholic steatohepatitis and prediabetes or type 2 diabetes mellitus. Ann Internal Med. (2016) 165:305–15. doi: 10.7326/M15-1774. PMID: 27322798

[17] Loomba R Sanyal AJ Nakajima A Neuschwander-Tetri BA Goodman ZD Harrison SA . Pegbelfermin in patients with nonalcoholic steatohepatitis and stage 3 fibrosis (FALCON 1): A randomized phase 2b study. Clin Gastroenterol Hepatol. (2024) 22:102–12. doi: 10.1016/j.cgh.2023.04.011. PMID: 37088457

[18] Harrison SA Wong VWS Okanoue T Bzowej N Vuppalanchi R Younes Z . Selonsertib for patients with bridging fibrosis or compensated cirrhosis due to NASH: Results from randomized phase III STELLAR trials. J Hepatol. (2020) 73:26–39. doi: 10.1016/j.jhep.2020.02.027. PMID: 32147362

[19] Harrison SA Bashir MR Guy CD Zhou R Moylan CA Frias JP . Resmetirom (MGL-3196) for the treatment of non-alcoholic steatohepatitis: a multicentre, randomised, double-blind, placebo-controlled, phase 2 trial. Lancet. (2019) 394:2012–24. doi: 10.1016/S0140-6736(19)32517-6. PMID: 31727409

[20] Harrison SA Alkhouri N Davison BA Sanyal A Edwards C Colca JR . Insulin sensitizer MSDC-0602K in non-alcoholic steatohepatitis: A randomized, double-blind, placebo-controlled phase IIb study. J Hepatol. (2020) 72:613–26. doi: 10.1016/j.jhep.2019.10.023. PMID: 31697972

[21] Harrison SA Neff G Guy CD Bashir MR Paredes AH Frias JP . Efficacy and safety of aldafermin, an engineered FGF19 analog, in a randomized, double-blind, placebo-controlled trial of patients with nonalcoholic steatohepatitis. Gastroenterology. (2021) 160:219–31. doi: 10.1053/j.gastro.2020.08.004. PMID: 32781086

[22] Okanoue T Sakamoto M Harada K Inagaki M Totsuka N Hashimoto G . Efficacy and safety of apararenone (MT-3995) in patients with nonalcoholic steatohepatitis: A randomized controlled study. Hepatol Res. (2021) 51:943–56. doi: 10.1111/hepr.13695. PMID: 34260795

[23] Friedman SL Ratziu V Harrison SA Abdelmalek MF Aithal GP Caballeria J . A randomized, placebo-controlled trial of cenicriviroc for treatment of nonalcoholic steatohepatitis with fibrosis. Hepatology. (2018) 67:1754–67. doi: 10.1002/hep.29477. PMID: 28833331 PMC5947654

[24] Harrison SA Ruane PJ Freilich B Neff G Patil R Behling C . A randomized, double-blind, placebo-controlled phase IIa trial of efruxifermin for patients with compensated NASH cirrhosis. JHEP Rep. (2022) 5:100563. doi: 10.1016/j.jhepr.2022.100563. PMID: 36644237 PMC9832280

[25] Harrison SA Goodman Z Jabbar A Vemulapalli R Younes ZH Freilich B . A randomized, placebo-controlled trial of emricasan in patients with NASH and F1-F3 fibrosis. J Hepatol. (2020) 72:816–27. doi: 10.1016/j.jhep.2019.11.024. PMID: 31887369

[26] Harrison SA Frias JP Lucas KJ Reiss G Neff G Bollepalli S . Safety and efficacy of efruxifermin in combination with a GLP-1 receptor agonist in patients with NASH/MASH and type 2 diabetes in a randomized phase 2 study. Clin Gastroenterol Hepatol. (2025) 23:103–13. doi: 10.1016/j.cgh.2024.02.022. PMID: 38447814

[27] Sanyal AJ Bedossa P Fraessdorf M Neff GW Lawitz E Bugianesi E . A phase 2 randomized trial of survodutide in MASH and fibrosis. N Engl J Med. (2024) 391:311–9. doi: 10.1056/NEJMoa2401755. PMID: 38847460

[28] Francque SM Bedossa P Ratziu V Anstee QM Bugianesi E Sanyal AJ . A randomized, controlled trial of the pan-PPAR agonist lanifibranor in NASH. N Engl J Med. (2021) 385:1547–58. doi: 10.1056/NEJMoa2036205. PMID: 34670042

[29] Loomba R Noureddin M Kowdley KV Kohli A Sheikh A Neff G . Combination therapies including cilofexor and firsocostat for bridging fibrosis and cirrhosis due to NASH. Hepatology. (2021) 73:625–43. doi: 10.1002/hep.31622. PMID: 33169409

[30] Newsome PN Buchholtz K Cusi K Linder M Okanoue T Ratziu V . A placebo-controlled trial of subcutaneous semaglutide in nonalcoholic steatohepatitis. N Engl J Med. (2021) 384:1113–24. doi: 10.1056/NEJMoa2028395. PMID: 33185364

[31] Younossi ZM Ratziu V Loomba R Rinella M Anstee QM Goodman Z . Obeticholic acid for the treatment of non-alcoholic steatohepatitis: interim analysis from a multicentre, randomised, placebo-controlled phase 3 trial. Lancet. (2019) 394:2184–96. doi: 10.1016/S0140-6736(19)33041-7. PMID: 31813633

[32] Harrison SA Thang C Bolze S Grouin JM Moller DE Fouqueray P . Evaluation of PXL065–deuterium-stabilized (R)-pioglitazone in patients with NASH: A phase II randomized placebo-controlled trial (DESTINY-1). J Hepatol. (2023) 78:914–25. doi: 10.1016/j.jhep.2023.02.004. PMID: 36804402

[33] Chan WK Nik Mustapha NR Mahadeva S . A randomized trial of silymarin for the treatment of non-alcoholic steatohepatitis. Clin Gastroenterol Hepatol. (2017) 15:1940–1949.e8. doi: 10.1016/j.cgh.2017.04.016. PMID: 28419855

[34] Ratziu V Harrison S Francque S Bedossa P Lehert P Serfaty L . Elafibranor, an agonist of the peroxisome proliferator-activated receptor-α and -δ, induces resolution of nonalcoholic steatohepatitis without fibrosis worsening. Gastroenterology. (2016) 150:1147–1159.e5. doi: 10.1053/j.gastro.2016.01.038. PMID: 26874076

[35] Loomba R Bedossa P Grimmer K Kemble G Martins EB McCulloch W . Denifanstat for the treatment of metabolic dysfunction-associated steatohepatitis: a multicentre, double-blind, randomised, placebo-controlled, phase 2b trial. Lancet Gastroenterol Hepatol. (2024) 9:1090–100. doi: 10.1016/S2468-1253(24)00246-2. PMID: 39396529

[36] Sanyal AJ Newsome PN Kliers I Østergaard LH Long MT Kjær MS . Phase 3 trial of semaglutide in metabolic dysfunction–associated steatohepatitis. N Engl J Med. (2025) 392:2089–99. doi: 10.1056/NEJMoa2413258. PMID: 40305708

[37] Harrison SA Abdelmalek MF Neff G Gunn N Guy CD Alkhouri N . Aldafermin in patients with non-alcoholic steatohepatitis (ALPINE 2/3): a randomised, double-blind, placebo-controlled, phase 2b trial. Lancet Gastroenterol Hepatol. (2022) 7:603–16. doi: 10.1016/S2468-1253(22)00017-6. PMID: 35325622

[38] Hoofnagle JH Van Natta ML Kleiner DE Clark JM Kowdley KV Loomba R . Vitamin E and changes in serum alanine aminotransferase levels in patients with non-alcoholic steatohepatitis. Alimentary Pharmacol Ther. (2013) 38:134–43. doi: 10.1111/apt.12352. PMID: 23718573 PMC3775262

[39] Anstee QM Neuschwander-Tetri BA Wong VW Abdelmalek MF Rodriguez-Araujo G Landgren H . Cenicriviroc lacked efficacy to treat liver fibrosis in nonalcoholic steatohepatitis: AURORA phase III randomized study. Clin Gastroenterol Hepatol. (2024) 22:124–134.e1. doi: 10.1016/j.cgh.2023.04.003. PMID: 37061109

[40] Noureddin M Rinella ME Chalasani NP Neff GW Lucas KJ Rodriguez ME . Efruxifermin in compensated liver cirrhosis caused by MASH. N Engl J Med. (2025) 392:2413–24. doi: 10.1056/NEJMoa2502242. PMID: 40341827

