# Supplementary material for: Updating the MASH pharmacotherapy landscape: a network meta-analysis incorporating SGLT2 inhibitors and emerging combination therapies
Source: Front Endocrinol (Lausanne). 2026 Jun 4;17:1829315. doi: 10.3389/fendo.2026.1829315 (PMC13275242; doi:10.3389/fendo.2026.1829315)
Supplement: Supplementary file 1 [file DataSheet1.docx]

Supplementary Material

# Supplementary Figures and Tables

## Supplementary Figures

**Supplementary Figure S1.** Risk of bias summary plot. The stacked bar chart presents the review authors' overall judgements about each risk of bias domain, presented as percentages across all included randomized controlled trials.

**Supplementary Figure S2.** Detailed risk of bias assessment for each included study (traffic light plot). The plot illustrates the risk of bias judgements for each specific domain (D1-D5) and the overall risk of bias for the individual trials. Green circles with a plus sign (+) indicate a low risk of bias, yellow circles with a question mark (?) indicate some concerns, and red circles with a minus sign (-) indicate a high risk of bias. Domains: D1, Randomization process; D2, Deviations from intended interventions; D3, Missing outcome data; D4, Measurement of the outcome; D5, Selection of the reported result.
