# Supplementary material for: Updating the MASH pharmacotherapy landscape: a network meta-analysis incorporating SGLT2 inhibitors and emerging combination therapies
Source: Front Endocrinol (Lausanne). 2026 Jun 4;17:1829315. doi: 10.3389/fendo.2026.1829315 (PMC13275242; doi:10.3389/fendo.2026.1829315)
Supplement: Supplementary file 4 [file Table3.docx]

Supplementary Table S3.

| **Study (First Author, Year) [Trial Name]** | **NCT Identifier** | **Intervention (Mechanism Class)** | **Duration** | **Sample Size (Active/Placebo)** | **Fibrosis Stages Included** | **Network Contribution** |
| --- | --- | --- | --- | --- | --- | --- |
| Harrison, 2024 [MAESTRO-NASH] (1) | NCT03900429 | Resmetirom 80/100mg (THR-beta Agonist) | 52 weeks | 645 / 321 | F1B-F3 | Fibrosis + MASH resolution |
| Harrison, 2021 [BALANCED] (2) | NCT03976401 | Efruxifermin 28/50/70mg (FGF21 Analog) | 16 weeks | 59 / 21 | F1-F3 | Fibrosis + MASH resolution |
| Loomba, 2023 [ENLIVEN] (3) | NCT04929483 | Pegozafermin 15/30/44mg (FGF21 Analog) | 24 weeks | 151 / 71 | F1-F4 | Fibrosis + MASH resolution |
| Loomba, 2024 [SYNERGY-NASH] (4) | NCT04166773 | Tirzepatide 5/10/15mg (GLP-1/GIP Dual Agonist) | 52 weeks | 142 / 48 | F2-F3 | Fibrosis + MASH resolution |
| Lin, 2025 [DEAN trial] (5) | NCT03723252 | Dapagliflozin 10mg (SGLT2 Inhibitor) | 48 weeks | 78 / 76 | F1-F3 | Fibrosis + MASH resolution |
| Shankar, 2024 [PROXYMO] (6) | NCT04019561 | Cotadutide 300/600μg (GLP-1/Glucagon Dual Agonist) | 19 weeks | 50 / 24 | F1-F3 | Fibrosis + MASH resolution |
| Armstrong, 2016 [LEAN] (7) | NCT01237119 | Liraglutide 1.8mg (GLP-1 RA) | 48 weeks | 26 / 26 | F0-F4 | Fibrosis + MASH resolution |
| Harrison, 2023 [HARMONY] (8) | NCT04767529 | Efruxifermin 28/50mg (FGF21 Analog) | 24 weeks | 85 / 43 | F2-F3 | Fibrosis + MASH resolution |
| Cusi, 2016 [Long‑Term Pioglitazone for NASH] (9) | NCT00994682 | Pioglitazone 45mg (PPAR‑gamma Agonist) | 36 months | 50 / 51 | F0‑F3 | Fibrosis + MASH resolution |
| Loomba, 2024 [FALCON 1] (10) | NCT03486899 | Pegbelfermin 10/20/40mg (FGF21 Analog) | 48 weeks | 148 / 49 | F3 | Fibrosis + MASH resolution |
| Harrison, 2020 [STELLAR-3] (11) | NCT03053050 | Selonsertib 18/6mg (ASK1 Inhibitor) | 48 weeks | 643 / 159 | F3 | Fibrosis + MASH resolution |
| Harrison, 2020 [STELLAR-4] (11) | NCT03053063 | Selonsertib 18/6mg (ASK1 Inhibitor) | 48 weeks | 705 / 172 | F4 | Fibrosis + MASH resolution |
| Harrison, 2019 [MGL-3196-05] (12) | NCT02912260 | Resmetirom 80mg (THR-beta Agonist) | 36 weeks | 84 / 41 | F1-F3 | Fibrosis + MASH resolution |
| Harrison, 2020 [EMMINENCE] (13) | NCT02784444 | MSDC-0602K 62.5/125/250mg (MPC Modulator) | 52 weeks | 298 / 94 | F1-F3 | Fibrosis + MASH resolution |
| Harrison, 2020 [Aldafermin Phase II] (14) | NCT02443116 | Aldafermin 1mg (FGF19 Analog) | 24 weeks | 53 / 25 | F2-F3 | Fibrosis + MASH resolution |
| Okanoue, 2021 (15) | NR | Apararenone 10mg (MRA) | 72 weeks | 25 / 23 | F2-F3 | Fibrosis + MASH resolution |
| Friedman, 2018 [CENTAUR] (16) | NCT02217475 | Cenicriviroc 150mg (CCR2/5 Antagonist) | 48 weeks | 145 / 144 | F1-F3 | Fibrosis + MASH resolution |
| Harrison, 2022 [BALANCED-C] (17) | NCT03976401 | Efruxifermin 50mg (FGF21 Analog) | 16 weeks | 10 / NR | F4 | Safety and tolerability of efruxifermin |
| Harrison, 2020 (18) | NCT02686762 | Emricasan 5/50mg (Caspase Inhibitor) | 72 weeks | 213 / 105 | F1-F3 | Fibrosis + MASH resolution |
| Harrison, 2025 [Cohort D] (19) | NCT05039450 | Efruxifermin 50mg (FGF21 Analog) | 12 weeks | 10 / NR | F1-F3 | Safety and tolerability of efruxifermin + GLP1RA |
| Sanyal, 2024 (20) | NCT04771273 | Survodutide 2.4/4.8/6.0mg (GLP-1/Glucagon Dual Agonist) | 48 weeks | 219 / 74 | F1-F3 | Fibrosis + MASH resolution |
| Francque, 2021 [NATIVE] (21) | NCT03008070 | Lanifibranor 800/1200mg (Pan-PPAR Agonist) | 24 weeks | 166 / 81 | F0-F3 | Fibrosis + MASH resolution |
| Loomba, 2021 [ATLAS] (22) | NCT03449446 | Selo/Firso/Cilo ± Combinations (Combination Therapy) | 48 weeks | 312 / 78 | F3-F4 | Fibrosis + MASH resolution |
| Newsome, 2020 (23) | NCT02970942 | Semaglutide 0.1/0.2/0.4mg (GLP-1 RA) | 72 weeks | 240 / 80 | F1-F3 | Fibrosis + MASH resolution |
| Younossi, 2019 [REGENERATE] (24) | NCT02548351 | OCA 10/25mg (FXR Agonist) | 72 weeks | 620 / 311 | F2-F3 | Fibrosis + MASH resolution |
| Harrison, 2023 [DESTINY-1] (25) | NCT04321343 | PXL065 7.5/15/22.5mg (PPAR-gamma Agonist) | 36 weeks | 87 / 30 | F1-F3 | Fibrosis + MASH resolution |
| Chan, 2017 (26) | NCT02006498 | Silymarin 700mg TID (Antioxidant) | 48 weeks | 49 / 50 | F0-F3 | Fibrosis + MASH resolution |
| Ratziu, 2016 [GOLDEN-505] (27) | NCT01694849 | Elafibranor 80/120mg (Pan-PPAR Agonist) | 52 weeks | 184 / 92 | F0-F3 | Fibrosis + MASH resolution |
| Loomba, 2024 [FASCINATE-2] (28) | NCT04906421 | Denifanstat 50mg (FASN Inhibitor) | 52 weeks | 112 / 56 | F2-F3 | Fibrosis + MASH resolution |
| Sanyal, 2025 [ESSENCE] (29) | NCT04822181 | Semaglutide 2.4mg (GLP-1 RA) | 72 weeks | 534 / 266 | F2-F3 | Fibrosis + MASH resolution |
| Harrison, 2022 [ALPINE 2/3] (30) | NCT03912532 | Aldafermin 0.3/1.0/3.0mg (FGF19 Analog) | 24 weeks | 128 / 43 | F2-F3 | Fibrosis + MASH resolution |
| Hoofnagle, 2013 [PIVENS] (31) | NCT00063622 | Vitamin E 800IU (Antioxidant) | 96 weeks | 71 / 68 | Mean stage 1.5-1.6 | Fibrosis + MASH resolution |
| Anstee, 2024 [AURORA] (32) | NCT03028740 | Cenicriviroc 150mg (CCR2/5 Antagonist) | 52 weeks | 1185 / 593 | F2-F3 | Fibrosis + MASH resolution |
| Noureddin, 2025 [SYMMETRY] (33) | NCT05039450 | Efruxifermin 28/50mg (FGF21 Analog) | 96 weeks | 120 / 61 | F4 | Fibrosis + MASH resolution |

**References**

1. Harrison SA, Bedossa P, Guy CD, Schattenberg JM, Loomba R, Taub R, et al. A phase 3, randomized, controlled trial of resmetirom in NASH with liver fibrosis. New England Journal of Medicine. (2024) 390(6):497-509. doi: 10.1056/NEJMoa2309000.
2. Harrison SA, Ruane PJ, Freilich BL, Neff G, Patil R, Behling CA, et al. Efruxifermin in non-alcoholic steatohepatitis: a randomized, double-blind, placebo-controlled, phase 2a trial. Nature Medicine. (2021) 27(7):1262-1271. doi: 10.1038/s41591-021-01425-3.
3. Loomba R, Sanyal AJ, Kowdley KV, Bhatt DL, Alkhouri N, Frias JP, et al. Randomized, controlled trial of the FGF21 analogue pegozafermin in NASH. New England Journal of Medicine. (2023) 389(11):998-1008. doi: 10.1056/NEJMoa2304286.
4. Loomba R, Hartman ML, Lawitz EJ, Vuppalanchi R, Boursier J, Bugianesi E, et al. Tirzepatide for metabolic dysfunction–associated steatohepatitis with liver fibrosis. New England Journal of Medicine. (2024) 391(4):299-310. doi: 10.1056/NEJMoa2401943.
5. Lin J, Huang Y, Xu B, Gu X, Huang J, Sun J, et al. Effect of dapagliflozin on metabolic dysfunction-associated steatohepatitis: a multicentre, double-blind, randomised, placebo-controlled trial. BMJ. (2025) 389:e083735. doi: 10.1136/bmj-2024-083735.
6. Shankar SS, Daniels SJ, Robertson D, Sarv J, Sánchez J, Carter D, et al. Safety and Efficacy of Novel Incretin Co-agonist Cotadutide in Biopsy-proven Noncirrhotic MASH With Fibrosis. Clinical Gastroenterology and Hepatology. (2024) 22(9):1847-1857. doi: 10.1016/j.cgh.2024.04.017.
7. Armstrong MJ, Gaunt P, Aithal GP, Barton D, Hull D, Parker R, et al. Liraglutide safety and efficacy in patients with non-alcoholic steatohepatitis (LEAN): a multicentre, double-blind, randomised, placebo-controlled phase 2 study. The Lancet. (2016) 387(10019):679-690. doi: 10.1016/S0140-6736(15)00803-X.
8. Harrison SA, Frias JP, Neff G, Abrams GA, Lucas KJ, Sanchez W, et al. Safety and efficacy of once-weekly efruxifermin versus placebo in non-alcoholic steatohepatitis (HARMONY): a multicentre, randomised, double-blind, placebo-controlled, phase 2b trial. The Lancet Gastroenterology & Hepatology. (2023) 8(12):1080-1093. doi: 10.1016/S2468-1253(23)00272-8.
9. Cusi K, Orsak B, Bril F, Lomonaco R, Hecht J, Ortiz-Lopez C, et al. Long-Term Pioglitazone Treatment for Patients With Nonalcoholic Steatohepatitis and Prediabetes or Type 2 Diabetes Mellitus. Annals of Internal Medicine. (2016) 165(5):305-315. doi: 10.7326/M15-1774.
10. Loomba R, Sanyal AJ, Nakajima A, Neuschwander-Tetri BA, Goodman ZD, Harrison SA, et al. Pegbelfermin in Patients With Nonalcoholic Steatohepatitis and Stage 3 Fibrosis (FALCON 1): A Randomized Phase 2b Study. Clinical Gastroenterology and Hepatology. (2024) 22(1):102-112. doi: 10.1016/j.cgh.2023.04.011.
11. Harrison SA, Wong VWS, Okanoue T, Bzowej N, Vuppalanchi R, Younes Z, et al. Selonsertib for patients with bridging fibrosis or compensated cirrhosis due to NASH: Results from randomized phase III STELLAR trials. Journal of Hepatology. (2020) 73(1):26-39. doi: 10.1016/j.jhep.2020.02.027.
12. Harrison SA, Bashir MR, Guy CD, Zhou R, Moylan CA, Frias JP, et al. Resmetirom (MGL-3196) for the treatment of non-alcoholic steatohepatitis: a multicentre, randomised, double-blind, placebo-controlled, phase 2 trial. The Lancet. (2019) 394(10213):2012-2024. doi: 10.1016/S0140-6736(19)32517-6.
13. Harrison SA, Alkhouri N, Davison BA, Sanyal A, Edwards C, Colca JR, et al. Insulin sensitizer MSDC-0602K in non-alcoholic steatohepatitis: A randomized, double-blind, placebo-controlled phase IIb study. Journal of Hepatology. (2020) 72(4):613-626. doi: 10.1016/j.jhep.2019.10.023.
14. Harrison SA, Neff G, Guy CD, Bashir MR, Paredes AH, Frias JP, et al. Efficacy and Safety of Aldafermin, an Engineered FGF19 Analog, in a Randomized, Double-Blind, Placebo-Controlled Trial of Patients With Nonalcoholic Steatohepatitis. Gastroenterology. (2021) 160(1):219-231. doi: 10.1053/j.gastro.2020.08.004.
15. Okanoue T, Sakamoto M, Harada K, Inagaki M, Totsuka N, Hashimoto G, et al. Efficacy and safety of apararenone (MT-3995) in patients with nonalcoholic steatohepatitis: A randomized controlled study. Hepatology Research. (2021) 51(9):943-956. doi: 10.1111/hepr.13695.
16. Friedman SL, Ratziu V, Harrison SA, Abdelmalek MF, Aithal GP, Caballeria J, et al. A Randomized, Placebo-Controlled Trial of Cenicriviroc for Treatment of Nonalcoholic Steatohepatitis With Fibrosis. Hepatology. (2018) 67(5):1754-1767. doi: 10.1002/hep.29477.
17. Harrison SA, Ruane PJ, Freilich B, Neff G, Patil R, Behling C, et al. A randomized, double-blind, placebo-controlled phase IIa trial of efruxifermin for patients with compensated NASH cirrhosis. JHEP Reports. (2022) 5(1):100563. doi: 10.1016/j.jhepr.2022.100563.
18. Harrison SA, Goodman Z, Jabbar A, Vemulapalli R, Younes ZH, Freilich B, et al. A randomized, placebo-controlled trial of emricasan in patients with NASH and F1-F3 fibrosis. Journal of Hepatology. (2020) 72(5):816-827. doi: 10.1016/j.jhep.2019.11.024.
19. Harrison SA, Frias JP, Lucas KJ, Reiss G, Neff G, Bollepalli S, et al. Safety and Efficacy of Efruxifermin in Combination With a GLP-1 Receptor Agonist in Patients With NASH/MASH and Type 2 Diabetes in a Randomized Phase 2 Study. Clinical Gastroenterology and Hepatology. (2025) 23(1):103-113. doi: 10.1016/j.cgh.2024.02.022.
20. Sanyal AJ, Bedossa P, Fraessdorf M, Neff GW, Lawitz E, Bugianesi E, et al. A Phase 2 Randomized Trial of Survodutide in MASH and Fibrosis. New England Journal of Medicine. (2024) 391(4):311-319. doi: 10.1056/NEJMoa2401755.
21. Francque SM, Bedossa P, Ratziu V, Anstee QM, Bugianesi E, Sanyal AJ, et al. A Randomized, Controlled Trial of the Pan-PPAR Agonist Lanifibranor in NASH. New England Journal of Medicine. (2021) 385(17):1547-1558. doi: 10.1056/NEJMoa2036205.
22. Loomba R, Noureddin M, Kowdley KV, Kohli A, Sheikh A, Neff G, et al. Combination therapies including cilofexor and firsocostat for bridging fibrosis and cirrhosis due to NASH. Hepatology. (2021) 73(2):625-643. doi: 10.1002/hep.31622.
23. Newsome PN, Buchholtz K, Cusi K, Linder M, Okanoue T, Ratziu V, et al. A Placebo-Controlled Trial of Subcutaneous Semaglutide in Nonalcoholic Steatohepatitis. New England Journal of Medicine. (2021) 384(12):1113-1124. doi: 10.1056/NEJMoa2028395.
24. Younossi ZM, Ratziu V, Loomba R, Rinella M, Anstee QM, Goodman Z, et al. Obeticholic acid for the treatment of non-alcoholic steatohepatitis: interim analysis from a multicentre, randomised, placebo-controlled phase 3 trial. The Lancet. (2019) 394(10215):2184-2196. doi: 10.1016/S0140-6736(19)33041-7.
25. Harrison SA, Thang C, Bolze S, Grouin JM, Moller DE, Fouqueray P. Evaluation of PXL065–deuterium-stabilized (R)-pioglitazone in patients with NASH: A phase II randomized placebo-controlled trial (DESTINY-1). Journal of Hepatology. (2023) 78(5):914-925. doi: 10.1016/j.jhep.2023.02.004.
26. Chan WK, Nik Mustapha NR, Mahadeva S. A Randomized Trial of Silymarin for the Treatment of Non-alcoholic Steatohepatitis. Clinical Gastroenterology and Hepatology. (2017) 15(12):1940-1949.e8. doi: 10.1016/j.cgh.2017.04.016.
27. Ratziu V, Harrison S, Francque S, Bedossa P, Lehert P, Serfaty L, et al. Elafibranor, an Agonist of the Peroxisome Proliferator-activated Receptor-α and -δ, Induces Resolution of Nonalcoholic Steatohepatitis Without Fibrosis Worsening. Gastroenterology. (2016) 150(5):1147-1159.e5. doi: 10.1053/j.gastro.2016.01.038.
28. Loomba R, Bedossa P, Grimmer K, Kemble G, Martins EB, McCulloch W, et al. Denifanstat for the treatment of metabolic dysfunction-associated steatohepatitis: a multicentre, double-blind, randomised, placebo-controlled, phase 2b trial. The Lancet Gastroenterology & Hepatology. (2024) 9(12):1090-1100. doi: 10.1016/S2468-1253(24)00246-2.
29. Sanyal AJ, Newsome PN, Kliers I, Østergaard LH, Long MT, Kjær MS, et al. Phase 3 Trial of Semaglutide in Metabolic Dysfunction–Associated Steatohepatitis. New England Journal of Medicine. (2025) 392(21):2089-2099. doi: 10.1056/NEJMoa2413258.
30. Harrison SA, Abdelmalek MF, Neff G, Gunn N, Guy CD, Alkhouri N, et al. Aldafermin in patients with non-alcoholic steatohepatitis (ALPINE 2/3): a randomised, double-blind, placebo-controlled, phase 2b trial. The Lancet Gastroenterology & Hepatology. (2022) 7(7):603-616. doi: 10.1016/S2468-1253(22)00017-6.
31. Hoofnagle JH, Van Natta ML, Kleiner DE, Clark JM, Kowdley KV, Loomba R, et al. Vitamin E and changes in serum alanine aminotransferase levels in patients with non-alcoholic steatohepatitis. Alimentary Pharmacology & Therapeutics. (2013) 38(2):134-143. doi: 10.1111/apt.12352.
32. Anstee QM, Neuschwander-Tetri BA, Wong VW, Abdelmalek MF, Rodriguez-Araujo G, Landgren H, et al. Cenicriviroc Lacked Efficacy to Treat Liver Fibrosis in Nonalcoholic Steatohepatitis: AURORA Phase III Randomized Study. Clinical Gastroenterology and Hepatology. (2024) 22(1):124-134.e1. doi: 10.1016/j.cgh.2023.04.003.
33. Noureddin M, Rinella ME, Chalasani NP, Neff GW, Lucas KJ, Rodriguez ME, et al. Efruxifermin in Compensated Liver Cirrhosis Caused by MASH. New England Journal of Medicine. (2025) 392:2413-24. doi: 10.1056/NEJMoa2502242.
